# Perception of experience influences altruism and perception of agency influences trust in human–machine interactions

**DOI:** 10.1038/s41598-024-63360-w

**Published:** 2024-05-30

**Authors:** Mayada Oudah, Kinga Makovi, Kurt Gray, Balaraju Battu, Talal Rahwan

**Affiliations:** 1 Social Science Division, New York University Abu Dhabi, Abu Dhabi, UAE; 2 Department of Psychology and Neuroscience, University of North Carolina, Chapel Hill, USA; 3 Computer Science, Science Division, New York University Abu Dhabi, Abu Dhabi, UAE

**Keywords:** Psychology, Human behaviour

## Abstract

As robots become increasingly integrated into social economic interactions, it becomes crucial to understand how people perceive a robot’s mind. It has been argued that minds are perceived along two dimensions: experience, i.e., the ability to feel, and agency, i.e., the ability to act and take responsibility for one’s actions. However, the influence of these perceived dimensions on human–machine interactions, particularly those involving altruism and trust, remains unknown. We hypothesize that the perception of experience influences altruism, while the perception of agency influences trust. To test these hypotheses, we pair participants with bot partners in a dictator game (to measure altruism) and a trust game (to measure trust) while varying the bots’ perceived experience and agency, either by manipulating the degree to which the bot resembles humans, or by manipulating the description of the bots’ ability to feel and exercise self-control. The results demonstrate that the money transferred in the dictator game is influenced by the perceived experience, while the money transferred in the trust game is influenced by the perceived agency, thereby confirming our hypotheses. More broadly, our findings support the specificity of the mind hypothesis: Perceptions of different dimensions of the mind lead to different kinds of social behavior.

## Introduction

The onset of AI-powered human–machine interaction provides a chance to fundamentally transform society^1–3^. No longer confined to their traditional roles within factories, robots are now penetrating various spheres, including education^4^, elderly care^5^, and rehabilitation^6^. In traditional scenarios such as factories, robots often retain their identity as efficient tools. However, when operating in scenarios such as education or companionship for the elderly, people perceive robots differently—potentially as collaborative partners or friends^7^. This juxtaposition highlights the evolving nature of human–machine relationships and the nuanced roles robots assume in different contexts.

Amidst the emerging scenarios of human–machine interaction, there arises a pressing need for robots to transcend their role as mere tools and embrace their new role as collaborative partners^1,8^. The intricate fabric of human social interactions, interwoven with threads of altruism and trust, establishes a unique benchmark for these machines^9,10^. Altruism and trust are the foundational pillars of human society, with altruism embodying our moral compass^11,12^ and trust underpinning our ability to confidently navigate uncertainty^10,13^. Understanding how altruism and trust materialize in our interactions with robots assumes a paramount role in cultivating a harmonious coexistence between humans and robotic entities^14^.

Altruism and trust rely on how one perceives the mind of one’s interaction partner; the former requires empathizing with the other, i.e., perceiving their emotional state^15–18^, while the latter requires perceiving the others’ capacity to reciprocate^19,20^. The seminal work of Gray and Wegner^21–25^ demonstrated that people perceive the minds of others along two dimensions. The first dimension is experience, i.e., the capacity to feel emotions, while the second dimension is agency, i.e., the capacity to plan and act. For instance, adults are perceived as having full-blown experience and agency, while children are perceived as having moderate experience but limited agency^21,23,26^. Machines, on the other hand, are typically perceived to have almost no experience but some level of agency^21^. People may also perceive the mind of the same entity differently. For instance, children, but not adults, perceive toys as having experience^27^. Similarly, the way vegetarians perceive the mind of an animal differs from the way meat eaters perceive it^28^. Despite numerous studies that examine the perception of agency and experience^22–24,29,30^, the role that these two dimensions play in altruism and trust is still unknown.

We hypothesize that the perception of experience in an interaction partner predicts acting altruistically towards them. This idea is motivated by previous findings that altruism is influenced by empathy^17,18,31,32^, and that empathy is associated with the ability to perceive the emotional states of others^15,33^. Another hypothesis we put forward is that the perception of agency in an interaction partner predicts trust towards them. This idea is rooted in the fact that trust hinges on evaluating an individual’s capacity to reciprocate, which, in turn, relies on assessing their ability to plan and take action^34,35^. These two hypotheses make sense, bearing in mind that people are most concerned with the *experience* of those who are recipients of kindness or harm (referred to as moral “patients”), but most concerned with the *agency* of those who perform acts of kindness or harm (referred to as moral “agents”). To put it differently, recipients of altruism are those who are capable of receiving help, i.e., they are moral patients, and hence their perceived experience matters. On the other hand, recipients of trust are those who are capable of helping us (if they reciprocate) or harming us (if they choose not to), i.e., they are moral agents, and hence their perceived agency matters^22^.

To test these hypotheses, we need to measure people’s altruism and trust towards machines while varying the machine’s perceived experience and agency. To this end, we employ two canonical games: a one-shot Dictator Game (DG) and a one-shot Trust Game (TG). The DG involves two players: an allocator and a receiver. The allocator is given an endowment and gets to choose how much money (if any) to give to the receiver, while the receiver has no decision-making role^36,37^. Given that the allocator is not obliged to share any amount, and is not expecting any reciprocity, the amount they share can serve as a proxy measure for their altruism or prosocial behavior^37^. The TG is similar, except that, after the allocator decides how much to share, the shared amount gets tripled, and the receiver gets to choose how much (if any) to return to the allocator. Here, if the allocator fully trusts the receiver to share the tripled amount equally, then they are better off sharing their entire endowment. On the other extreme, if they fully distrust the receiver, they would keep the entire endowment to themselves. As such, the amount sent reflects the allocator’s assessment of the receiver’s trustworthiness^31,36–38^.

Based on the above, if we get participants to play the DG and TG with bot receivers, we would be able to measure people’s altruism and trust towards machines. The only remaining component in our experimental design is to vary the bot’s perceived experience and agency, to determine whether this affects people’s altruism and trust. To this end, we display to the participants an image representing the bot receiver, and we vary this image along a spectrum ranging from extremely machine-like to extremely human-like appearances. Here, the underlying hypothesis (which we test) is that bots are perceived as having greater agency and experience when they appear more human-like. The idea of anthropomorphizing robots is inspired by the work of the robotics pioneer, Mori^39^. In particular, Mori proposed that, as a robot’s appearance evolves from a machine-like form to a human-like one, the level of affinity for the robot tends to increase gradually. However, an intriguing exception arises when the robot’s appearance is almost (but not quite) human-like. In this case, the robot’s appearance starts eliciting eerie and repelling feelings, resulting in a sharp drop in affinity. This drop is widely known as “the uncanny valley,” due to the fact that, when plotting affinity against human-likeness, the drop creates a valley-like shape. Mori’s groundbreaking insight has since spurred numerous investigations into how a robot’s appearance affects those interacting with it^1,2,9,30,40–43^. We contribute to this literature, since our experimental design naturally allows us to study how the uncanny valley influences altruism, trust, and the perception of both agency and experience. More importantly, however, our design also allows us to examine how mind perception influences trust and altruism in human–machine interaction.

## Results

To generate our stimuli, we require a spectrum of bot images that exhibited varying levels of humanness, which we hypothesize would manipulate perceptions of agency and experience. Using deep learning techniques^44^, we generated five different spectra that are meant to range from extremely machine-like to extremely human-like; for a detailed description of how these were generated, see Supplementary Note 1. It should be noted that any of these spectra would reproduce the uncanny valley phenomenon if it satisfies the following two conditions: (i) the images become increasingly human-like; (ii) the images become increasingly pleasant, with the exception of a single image falling somewhere around the middle of the spectrum for which pleasantness drops notably.

**Figure 1:**
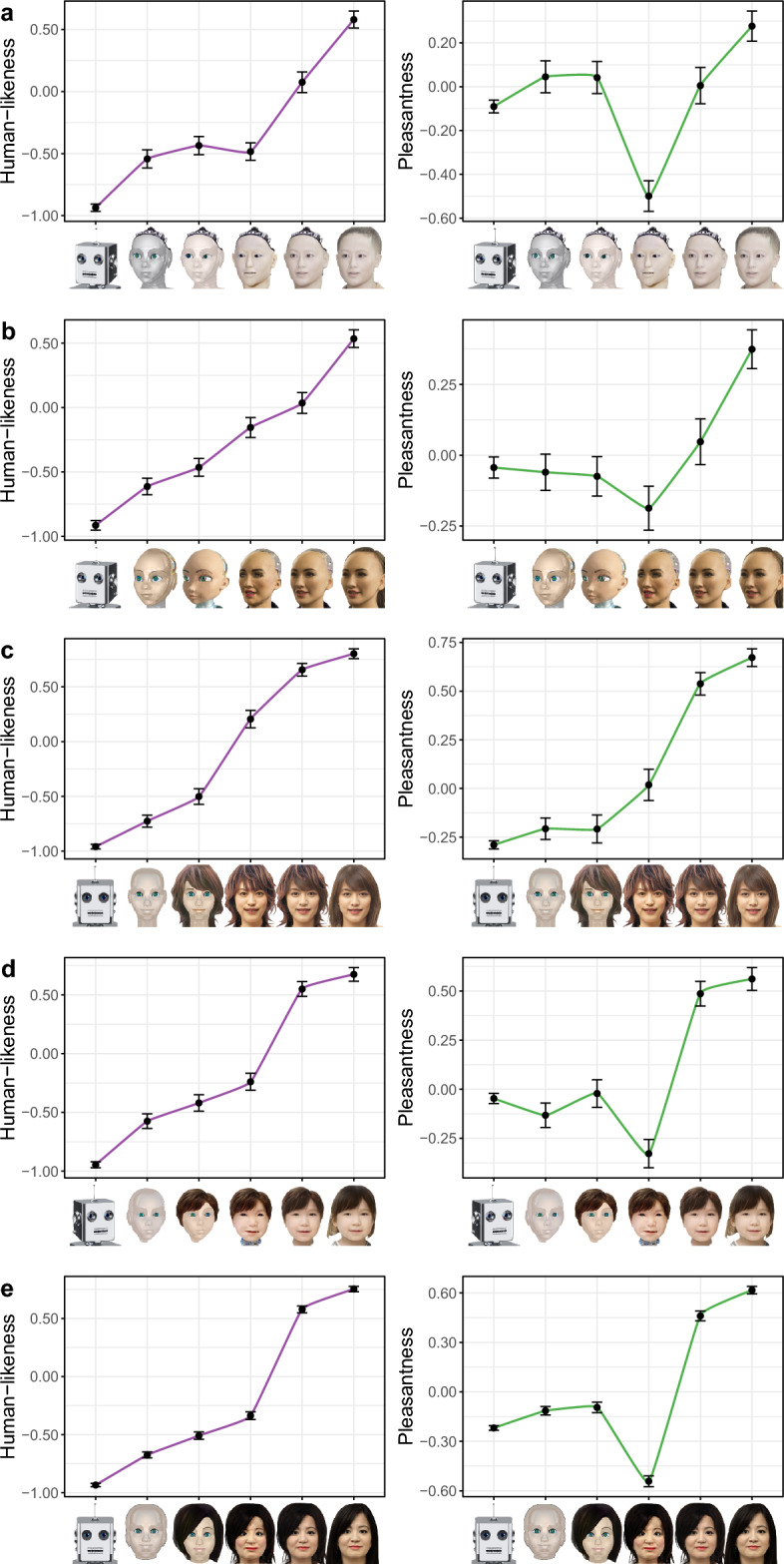
Perceived human-likeness and pleasantness across spectra. Each row corresponds to a different spectrum. Each x-axis depicts a spectrum of images that are meant to range from extremely machine-like to extremely human-like. The y-axes depict participants’ evaluation of the image’s human-likeness (left column) and pleasantness (right column). Error bars represent 95% confidence intervals.

In our first study (hereafter Study 1) for each of the five spectra, we asked 200 participants to evaluate each image therein based on human-likeness and pleasantness, amounting to 1000 participants in total. The results of this evaluation are depicted in Fig. 1. As can be seen, some spectra do not satisfy the above two conditions of the uncanny valley. For instance, the spectrum corresponding to Fig. 1a satisfies condition (ii) but not (i), i.e., the pleasantness curve produces a shape that resembles a valley—the uncanny valley—but the human-likeness does not increase monotonically. On the other hand, the spectrum corresponding to Fig. 1c satisfies condition (i) but not (ii), i.e., the images are increasingly human-like, but there is no dip in pleasantness around the middle of the spectrum. As for the spectrum corresponding to Fig. 1b, it does satisfy both conditions, but the valley does not appear to be as deep as the ones observed in Fig. 1d,e. Out of the latter two, we selected the one depicted in Fig. 1e, since it was the one with the deeper valley. Such a spectrum allows us to examine not only the link between trust, altruism, and mind perception, but also how they are all related to the uncanny valley phenomenon.

Having selected a spectrum, we are now ready to use its constituent images as stimuli in our next behavioural experiment, hereafter Study 2. In particular, we recruited 730 participants who engaged in a one-shot Dictator Game (DG) and a one-shot Trust Game (TG) with bot associates that are each represented by a different image along the spectrum. Participants were informed that their associates are robots that have been programmed to interact socially with humans and are able to make decisions. After completing the DG and TG, participants were asked to evaluate each image along the two dimensions of mind perception; see the Supplementary Materials for more details.

**Figure 2:**
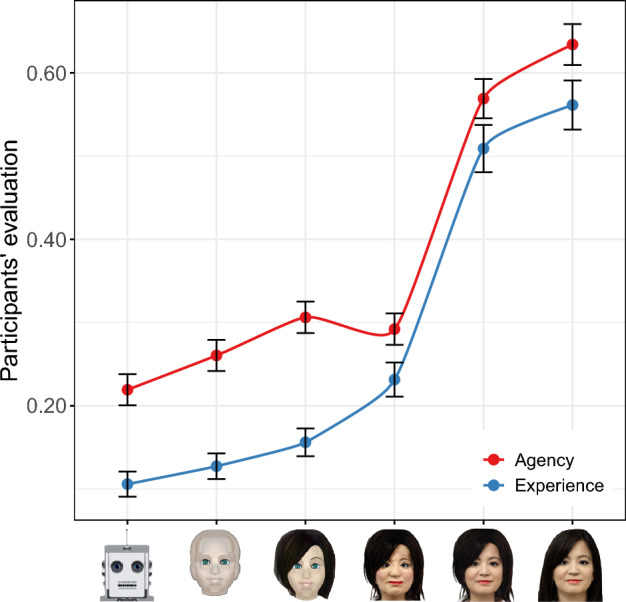
Perceived experience and agency across images. Participants’ perception of experience and agency given different images across our spectrum of choice. Error bars represent 95% confidence intervals.

Before presenting the DG and TG results, let us first present the participants’ evaluation of experience and agency. As shown in Fig. 2, the different images vary markedly along the two dimensions of mind perception, thereby confirming our first hypothesis—the perceived experience and agency of a robot can be manipulated by varying the degree to which that robot resembles a human. The overall trend in perceived experience resembles that of human-likeness, as evidenced by the correlation between the two (grouped by image), which is strong and statistically significant ($r = 0.99, p < 0.001$). On the other hand, the overall trend in perceived agency resembles that of pleasantness. Indeed, the correlation between the two (grouped by image) is strong and statistically significant ($r = 0.90, p = 0.014$).

The results in Fig. 2 contribute to our understanding of the uncanny valley phenomenon. Overall, the perceived experience and agency tend to increase with human-likeness. The only exception to this rule occurs at the uncanny image, where the notable increase in perceived experience (compared to the image before it in the spectrum) is associated with a slight reduction (rather than an increase) in perceived agency. In other words, judging from the overall trend, one would expect the perceived agency of the uncanny image to be greater, bearing in mind the notable increase in perceived experience. This discrepancy may explain the eerie and repelling feelings associated with the uncanny valley.

**Figure 3:**
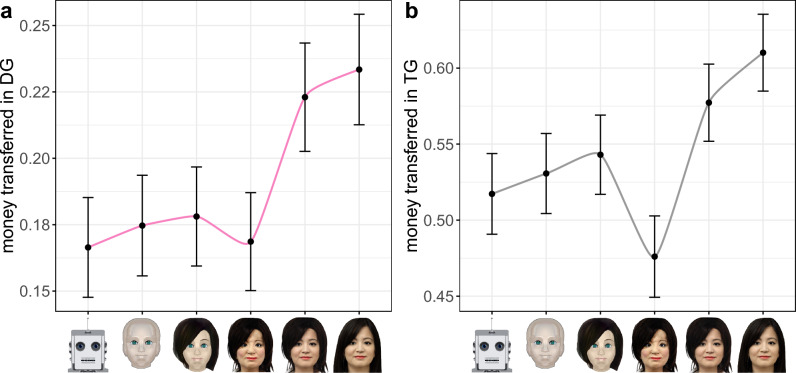
Money transferred in DG and TG across images. The amount of money participants transferred to their bot partner in a one-shot Dictator Game (**a**) and a one-shot Trust Game (**b**) given different images across the spectrum. Error bars represent 95% confidence intervals.

Having demonstrated that the images in our spectrum vary along the two dimensions of mind perception, we now use this spectrum to examine how the perception of experience and agency influences altruism and trust in human–machine interactions. To this end, we analyse the amount of money that participants transferred in both the DG (which serves as a proxy for altruism) as well as the TG (which serves as a proxy for trust) when paired with different images across the spectrum. The results for the DG and the TG are depicted in Fig. 3a,b, respectively.

A simple linear regression reveals that the perception of experience accounts for the variance observed in money transfers in the DG (adjusted $R^2 = 91\%$, $p = 0.002$, $\beta _1 = 0.141$). These findings support our hypothesis that perceiving experience in others is associated with showing altruism toward them. It should be noted that the average donation rate exceeds 20% when the bot image crosses the uncanny valley, making it comparable to the donation rates observed when interacting with humans in the DG^45^. Similarly, a simple linear regression reveals that the perception of agency accounts for most of the variance observed in money transfers in the TG (adjusted $R^2 = 65\%$, $p = 0.030$, $\beta _1 = 0.228$). These findings support our hypothesis that perceiving agency in others is associated with trusting them. Note that, apart from the uncanny image, the average donation rate exceeds 50%, which is greater than the rate observed when interacting with humans in the TG^46^. This observation is in agreement with previous findings in the literature, showing that people trust machines more than humans due to their betrayal aversion^47^. We measured the correlation between the amount of money transferred in both games, and found it to be weak ($r \le$0.26) regardless of the image used. Finally, we note that amount of money transferred in both games tends to increase with human-likeness, except for the uncanny image, where the amount drops, especially in the case of the TG.

So far, our results have shown that perceiving experience in bots is associated with acting altruistically toward them, whereas perceiving agency in bots is associated with trusting them. However, our findings are not sufficient to establish a causal link, since the images used in our experiment may differ in various attributes other than just their perceived agency and experience. For example, it could be the case that the bot images also vary along the dimension of attractiveness, with the perfectly human-like image being the most attractive, and the uncanny image being the least attractive. If true, then attractiveness may be a confounder that influences not only trust and altruism, but also the perception of agency and experience. More broadly, the associations observed in our study could be driven by an attribute (other than the dimensions of mind perception) that varies along our spectrum.

To rule out this possibility, we conducted a third behavioral experiment with 150 new participants, hereafter Study 3. This is similar to Study 2 except that, instead of manipulating the bot’s image, we now hold the image constant and explicitly manipulate the bot’s agency and experience using textual descriptions. In particular, the bot’s experience is manipulated by writing: “This robot is [capable/not capable] of feeling pain”, while the bot’s agency is manipulated by writing: “This robot is [capable/not capable] of planning actions and exercising self-control.” These descriptions were inspired by previous research on mind perception^29,30^. Consequently, we have four distinct conditions: (1) agency and experience; (2) agency but no experience; (3) experience but no agency; and (4) no agency nor experience. We repeated this experiment using two different bot images: the second and the second to last in our spectrum. see Supplementary Note 4 for more details.

**Figure 4:**
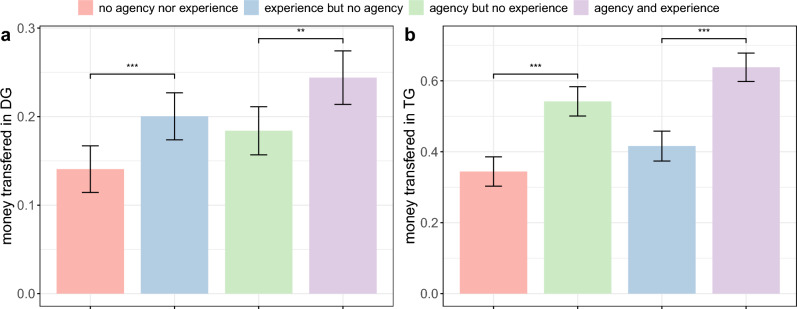
Money transferred in DG and TG in Study 3. The amount of money participants transferred to their bot partner in a one-shot Dictator Game (**a**) and a one-shot Trust Game (**b**) while explicitly varying the bot’s agency and experience. Error bars represent 95% confidence intervals. *p*-Value is calculated using Dunn test; ^∗^*p*<0.1; ^∗∗^*p*<0.05; ^∗∗∗^*p*<0.01..

We first run the Kruskal-Wallis H test to evaluate whether manipulating the bot’s agency and experience influences the contributions made in DG and TG while controlling for the bot’s image. We find that the two bot images have no effect on the contributions made in the DG ($p = 0.76$) and the TG ($p = 0.37$). To put it differently, the explicit manipulation of agency and experience through textual descriptions nullifies the effect of the bot image, which was previously observed in Study 2. Additionally, we find that the contributions made in DG and TG differ significantly across the four conditions of Study 3 ($p < 0.001$). Figure 4a,b depict the amount contributed in the DG and the TG, respectively, given each condition. As can be seen in Fig. 4a, participants act more altruistically toward bots that are described as having experience, regardless of whether they have agency. These findings provide causal evidence that the perception of experience influences altruism. Similarly, as shown in Fig. 4b, participants show greater trust in bots that are described as having agency, regardless of whether these bots have experience. These findings provide causal evidence that the perception of agency influences trust.

## Discussion

Our goal was to examine how mind perception influences trust and altruism in human–machine interaction. We conducted an experiment whereby people engage with bots in a one-shot Dictator Game (DG) and a one-shot Trust Game (TG) while varying the bot’s image along a spectrum ranging from extremely machine-like to extremely human-like appearances. We conducted another experiment whereby, instead of manipulating the bot’s image, we explicitly manipulated the bot’s agency and experience using textual description. The results of these experiments provide causal evidence in support of our main hypotheses, i.e., that the perception of experience influences altruism and that the perception of agency influences trust in human–machine interaction. More broadly, our findings provide the first evidence for what we call the “specificity of mind” hypothesis: Perceptions of different dimensions of the mind lead to different kinds of social behavior.

Our findings shed new light on the underlying mechanism behind altruism, showing that it depends on perceived experience. These results suggest that, in applications where people are required to act altruistically towards machines, designers need to emphasize the machine’s perceived capacity to feel, e.g., by giving it facial expressions. Our findings also shed new light on the mechanism underlying trust, showing that it is influenced by the perceived agency of the trustee. Indeed, trusting a machine requires endowing it with the ability to have control over, and take responsibility for, its own action (characteristics of agency). One practical implication of these findings is that imbuing robots with a sense of agency is crucial to cultivating trust in them. For instance, in service sectors such as banking, customer care, and career advice, manufacturers may consider designing robots that resemble human adults, since they are perceived to have greater agency than children^21^.

Finally, let us comment on the uncanny valley. In the literature, there have been several explanations for this phenomenon^40,42,43^, but none of them examines the dimensions of mind perception. The only exception is the work of Gray and Wegner^30^ who showed that uncanny robots are perceived to have experience. This, in turn, makes people feel uneasy since they consider experience to be fundamentally lacking in machines. However, these findings were based on a comparison between just two robots: one with an uncanny appearance and another with a mechanical appearance. In contrast, when we examined a spectrum of robot images ranging from machine-like to human-like appearances, we found that the most human-like robots are perceived to have the greatest experience (Fig. 2) yet they are the farthest from the uncanny valley (Fig. 1e, right panel), suggesting that the valley cannot be explained by the perception of experience alone. Furthermore, when images transform from machine-like to human-like, their perceived agency and experience are expected to increase, which is indeed the case for all bot images in our spectrum apart from the uncanny one. For that image, contrary to expectations, we found that the perceived experience increases notably (compared to the image falling just before it in the spectrum), while the perceived agency actually decreases slightly. These findings offer new insights into the mechanism underlying the uncanny valley, suggesting for the first time that the perception of agency might play a role in this phenomenon.

Our study underscores the significance of mind perception in shaping human–machine interactions. Understanding the nuances behind when and why people trust and cooperate with machines has never been more crucial, given the growing integration of machines in social and economic interactions. Although past work has highlighted a general link between mind perception and morality, this research is the first to demonstrate that the perception of different dimensions of the mind leads to different kinds of social behavior. Our study also touched on emerging issues related to morality and social robots, including how people treat machines and their affective reaction to human-like robots. Soon, we may be faced with moral decisions about not only other people but also machines, and these findings help to understand our behavior when that day comes.

## Methods

### Experimental procedures and measures

We needed to generate multiple spectra of robot images, each ranging from extremely machine-like to extremely human-like, with an uncanny image falling somewhere in between. We searched the Web for real-life humanoid robot images that are described as “uncanny” and meet the criteria proposed by Mathur and Reichling^48^, requiring a full face to be displayed, from the top of the head down to the chin, with both eyes visible. We identified five such images^49–52^. For each image, we used deep learning to make the image progressively human-like, and a graphics editor to make the image progressively machine-like, thereby producing a spectrum that corresponds to that image, see Supplementary Note 1 for more details.

The purpose of Study 1 was to identify the spectrum that best reproduces the uncanny valley phenomenon. Upon reading the consent form, participants were randomly assigned to one of the five spectra, and were asked to evaluate each image in that spectrum, displayed in random order. More specifically, following the design of Mathur and Reichling^48^, participants evaluated each image on a scale from − 100 to +100 according to human-likeness, pleasantness, trustworthiness, and feelings toward the image, see Supplementary Note 2 for more details. Furthermore, to ensure that our participants were paying attention, we added to the survey three attention-check questions, asking participants to set the slider to a specific, randomly chosen value. This way, we were able to identify those who were setting the slider without paying attention to the question at hand. To encourage participants to pay attention, we informed them that they would be paid an extra bonus of $1.00 (in addition to the participation fee) if they answered all attention-check questions correctly. Those who failed were subsequently excluded from our analysis. Moreover, we did not allow individuals to participate more than once in the study, to ensure that each individual evaluated exactly one spectrum.

Based on the outcome of Study 1, we selected the spectrum that best satisfied the following conditions: (i) the images become increasingly human-like; (ii) the images become increasingly pleasant, with the exception of a single image—the uncanny image—for which pleasantness drops notably. With our spectrum of choice, we were ready to run Study 2, where participants engage in a one-shot Dictator Game (DG) and a one-shot Trust Game (TG) with bot associates that are each represented by a different image along that spectrum. Upon reading the description of each game, participants were asked three comprehension-check questions to make sure they understood the rules. To encourage careful attention, participants were informed that they will be paid an extra bonus (in addition to the participation fee) if they answered all three questions correctly on their first attempt. Those who failed were asked to read the description once again before making another attempt; this was repeated until they got all questions correct. To study the relationship between participants’ perception of the chosen spectrum and their behavior when interacting with it, we invited the same individuals who took part in Study 1 to participate in Study 2, following a within-person design. We informed them that their associates are robots that have been programmed to interact socially with humans and are able to make decisions. For each participant, we randomly selected one of the two games (DG or TG), and asked them to play the selected game with six associates, in random order, each corresponding to a different image along the spectrum. The same process was then repeated for the other game. After completing the experimental games, participants were asked to evaluate each image, displayed in random order, according to experience and agency, see Supplementary Note 3 for more details.

Since all of our experiments involved playing the DG and TG with robots, it is unclear whether our findings can be generalized to the context of human-human interaction. This is because people’s behaviour may differ depending on whether their partner is a robot or a human, e.g., in the iterated prisoner’s dilemma, people cooperate less when they are informed that their partner is a machine, regardless of the partner’s actual choices in the game^53^. However, such biases do not affect our analyses, since all our participants had robot partners (and they were explicitly informed about this fact), regardless of the game they were engaged in (DG or TG) and regardless of the bot image they were interacting with along the spectrum.

### Data collection

All studies were programmed in Qualtrics. Participants were recruited on Amazon Mechanical Turk using the services of CloudResearch (previously TurkPrime^54^). Only MTurk workers 18 years or older, located in the United States—as specified on their MTurk account and by their IP address—could see the HIT (Human Intelligence Task). To be eligible, workers also needed to have at least 100 HITs approved and an 85% approval rating. We also excluded workers from suspicious geolocations and those on the “universal exclude list,” both managed by CloudResearch. In addition to these filters, we only recruited CloudResearch Approved participants to enhance data quality, as these individuals have exhibited high levels of engagement and attention in prior tasks^55,56^. We also employed multiple measures to ensure once in a lifetime participation, such as organizing all HITs in a survey group, and using the ballot box stuffing option on Qualtrics.

Data collection for Study 1 took place between the 21${\textrm{st}}$ of April and the 21${\textrm{th}}$ of May, 2021. We aimed for 200 participants per spectrum, amounting to a total of 1000 participants. However, since we excluded those who failed to answer all attention questions correctly, we had to recruit additional participants, ending up with a total of 1109 participants, 1000 of which answered all questions correctly. Based on the outcome of Study 1, we selected the spectrum to be used in Study 2. We decided to recruit 300 additional individuals to take part in Study 1 and evaluate our spectrum of choice, yielding a total of 500 who evaluated that spectrum.

Data collection for Study 2 took place between the 3$${\textrm{rd}}$$ of October and the 22$${\textrm{nd}}$$ December, 2021. To this end, we invited the 500 participants who evaluated the chosen spectrum in Study 1 to take part in Study 2; only 253 individuals agreed to participate. To reach the desired sample size based on power analysis, we recruited 550 additional individuals to evaluate the chosen spectrum (as per Study 1) between the 10$${\textrm{th}}$$ and 14$${\textrm{th}}$$ of November, 2021. At least 1 week after their participation in Study 1, those individuals were invited to take part in Study 2. We incentivized them by offering a raffle of $50 Amazon gift vouchers to five randomly-chosen participants who complete Study 2. 477 out of the 550 agreed to participate, resulting in a total of 730 participants for Study 2.

Using the same platform and quality control criteria of recruiting participants in the earlier two studies, data collection for Study 3 took place between the 14${\textrm{th}}$ and the 15${\textrm{th}}$ March, 2024. We recruited 150 participants who did not participate in Study 1 and Study 2.

### Compensation

Study 1 participants received a $0.50 participation fee in addition to a $1.00 bonus given only to those who answered all three attention check questions correctly. The average compensation in Study 1 was $1.50 (for those who answered correctly), and the average completion time was 7.38 minutes, yielding an hourly rate of $12.20. Study 2 participants received a $1.50 participation fee. They also received a bonus based on their earnings in the DG and TG, in addition to the bonus they received if they answered all comprehension check questions correctly on their first attempt ($0.25 for each game). The average compensation in Study 2 was $4.50, and the average completion time was 15.42 minutes, yielding an hourly rate of $17.51 (excluding the Amazon gift vouchers). The average compensation in Study 3 was $3.41, and the average completion time was 16.87 minutes, yielding an hourly rate of $12.13.

### Sample composition

Participants who completed Study 1 were 57.57% female (all other participants identified as male or other), and 70.49% identified as White (all other participants identified as American Indian or Alaska Native, Asian or Asian American, Black or African American, Hispanic or Latino/a, Middle Eastern or North African, Other, or identified with multiple of these categories), with an average age of 38.71 (sd = 12.59). Participants who were invited to complete Study 2 were 55.75% female, and 73.56% identified as White, with an average age of 40.16 (sd = 12.91). Attrition rate between Study 1 and Study 2 was 30.5%. We compared those who returned for Study 2 to those who did not, we found no meaningful differences between the two samples in terms of gender, education, income, racial identification, and level of familiarity with the robot in our chosen spectrum, namely Saya, see Supplementary Tables 5 and 6. Participants who completed Study 3 were 52% female and 48% male with an average age of 43.70 (sd = 11.72) and 76.67% identified as White. With the exception of one participant, none of the participants in Study 3 was familiar with any of the images used in the study.

### Pre-registration

We pre-registered Studies 1 and 2 on both OSF (https://osf.io/a375c/) and AsPredicted (https://aspredicted.org/; AsPredicted #106712). The pre-registration on OSF took place prior to the analysis of the outcome data for Study 1; it focused primarily on evaluating the proposed Uncanny Valley spectra. In contrast, the pre-registration on AsPredicted took place prior to the analysis of the outcome data for Study 2; it focused on investigating the influence of mind perception on altruism and trust.

### Ethical approval

All of our protocols received IRB approval by the NYU Abu Dhabi Internal Review Board (#19-2021), and all participants provided informed consent online to take part in the studies. All methods were performed in accordance with the relevant guidelines and regulations.

### Supplementary Information

Supplementary Information.

## Data Availability

The data can be found at the following repository: https://osf.io/a375c/.
