# Supplementary material for: Perception of experience influences altruism and perception of agency influences trust in human–machine interactions
Source: Sci Rep. 2024 May 30;14:12410. doi: 10.1038/s41598-024-63360-w (PMC11136977; doi:10.1038/s41598-024-63360-w)
Supplement: Supplementary file 1 — Supplementary Information. [file 41598_2024_63360_MOESM1_ESM.pdf]

Supplementary Materials
Perception of experience influences altruism and perception of agency influences trust in human-machine interactions

Mayada Oudah[1], Kinga Makovi[1], Kurt Gray[2], Balaraju Battu[3], and Talal Rahwan[3]

[1]Social Science Division, New York University Abu Dhabi, UAE.
[2]Department of Psychology and Neuroscience, University of North Carolina, Chapel Hill, USA.
[3]Computer Science, Science Division, New York University Abu Dhabi, UAE.

Contents

Supplementary Tables **2**

Supplementary Note 1: Spectrum Creation **8**

Supplementary Note 2: Spectrum Evaluation **13**

Supplementary Note 3: Study 2 - Graphical user interface (GUI) **15**

Supplementary Note 4: Study 3 - Graphical user interface (GUI) **22**

Supplementary Tables

Supplementary Table 1: Regression Results - Gender

	Dependent variable:	
	Experience	Agency
	(1)	(2)
Gender (Male)	0.052***	0.016
	(0.017)	(0.017)
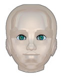	0.021*	0.041***
	(0.011)	(0.010)
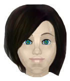	0.049***	0.087***
	(0.011)	(0.010)
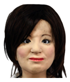	0.124***	0.073***
	(0.011)	(0.010)
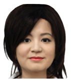	0.403***	0.351***
	(0.011)	(0.010)
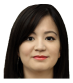	0.456***	0.416***
	(0.011)	(0.010)
Constant	0.083***	0.212***
	(0.013)	(0.013)
Observations	4,332	4,332
Log Likelihood	−203.274	175.606
Akaike Inf. Crit.	424.548	−333.212
Bayesian Inf. Crit.	481.912	−275.848
Note:	*p<0.1; **p<0.05; ***p<0.01	

Supplementary Table 2: Regression Results - Education

	Dependent variable:	
	Experience	Agency
	(1)	(2)
Education ($\geq$ Bachelor)	0.011	-0.040^{**}
	(0.017)	(0.017)
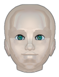	0.021^{*}	0.041^{***}
	(0.011)	(0.010)
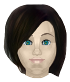	0.049^{***}	0.087^{***}
	(0.011)	(0.010)
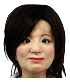	0.124^{***}	0.073^{***}
	(0.011)	(0.010)
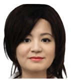	0.403^{***}	0.351^{***}
	(0.011)	(0.010)
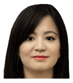	0.456^{***}	0.416^{***}
	(0.011)	(0.010)
Constant	0.100^{***}	0.240^{***}
	(0.014)	(0.014)
Observations	4,332	4,332
Log Likelihood	−207.890	178.123
Akaike Inf. Crit.	433.781	−338.245
Bayesian Inf. Crit.	491.145	−280.881
Note:	*p<0.1; **p<0.05; ***p<0.01	

Supplementary Table 3: Regression Results - Race

	Dependent variable:	
	Experience	Agency
	(1)	(2)
Race (White)	-0.051^{***}	-0.034^{*}
	(0.019)	(0.019)
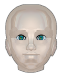	0.021^{*}	0.041^{***}
	(0.011)	(0.010)
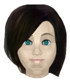	0.049^{***}	0.087^{***}
	(0.011)	(0.010)
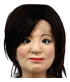	0.124^{***}	0.073^{***}
	(0.011)	(0.010)
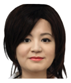	0.403^{***}	0.351^{***}
	(0.011)	(0.010)
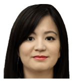	0.456^{***}	0.416^{***}
	(0.011)	(0.010)
Constant	0.143^{***}	0.244^{***}
	(0.018)	(0.017)
Observations	4,332	4,332
Log Likelihood	−204.345	176.936
Akaike Inf. Crit.	426.690	−335.872
Bayesian Inf. Crit.	484.054	−278.508
Note:	*p<0.1; **p<0.05; ***p<0.01	

Supplementary Table 4: Regression Results - Income

	Dependent variable:	
	Experience	Agency
	(1)	(2)
Income (≥ $60,000)	0.007	−0.008
	(0.017)	(0.017)
Income (Prefer not to say)	−0.055	−0.098*
	(0.051)	(0.051)
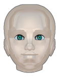	0.021*	0.041***
	(0.011)	(0.010)
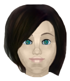	0.049***	0.087***
	(0.011)	(0.010)
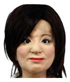	0.124***	0.073***
	(0.011)	(0.010)
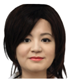	0.403***	0.351***
	(0.011)	(0.010)
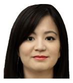	0.456***	0.416***
	(0.011)	(0.010)
Constant	0.104***	0.225***
	(0.014)	(0.013)
Observations	4,332	4,332
Log Likelihood	−209.404	174.974
Akaike Inf. Crit.	438.809	−329.948
Bayesian Inf. Crit.	502.547	−266.210
Note:	*p<0.1; **p<0.05; ***p<0.01	

	Study 1	Study 2	SMD
**N**	1850	730	
**Age** (%)			
Below 30	0.26 (0.44)	0.22 (0.42)	0.080
30 to 50	0.55 (0.50)	0.57 (0.50)	0.022
Above 50	0.19 (0.39)	0.21 (0.41)	0.058
**Education** (%)			
Less than Bachelor Degree	0.48 (0.50)	0.47 (0.50)	0.023
Bachelor Degree and above	0.52 (0.50)	0.53 (0.50)	0.023
**Ethnicity** (%)			
Other	0.30 (0.46)	0.27 (0.44)	0.053
White	0.70 (0.46)	0.73 (0.44)	0.053
**Income** (%)			
Less than $60,000	0.54 (0.50)	0.54 (0.50)	0.001
$60,000 and above	0.42 (0.49)	0.43 (0.50)	0.013
Prefer not to say	0.03 (0.18)	0.03 (0.17)	0.033
**Gender** (%)			
Male	0.42 (0.49)	0.44 (0.50)	0.036
Female	0.58 (0.49)	0.55 (0.50)	0.042
Other	0.01 (0.08)	0.01 (0.10)	0.035

Supplementary Table 5: **Sample composition of Studies 1 and 2.** The sample of Study 2 is restricted to participants from Study 1 who were assigned to Saya's spectrum and responded to our invitation to participate in Study 2. SMD stands for Standardized Mean Difference.

N	Study 1<br>1050	Study 1′<br>320	Study 2<br>730	SMD
**Age** (%)				
Below 30	0.27 (0.44)	0.37 (0.48)	0.22 (0.42)	0.212
30 to 50	0.55 (0.50)	0.51 (0.50)	0.57 (0.50)	0.075
Above 50	0.18 (0.39)	0.12 (0.33)	0.21 (0.41)	0.158
**Education** (%)				
Less than Bachelor Degree	0.51 (0.50)	0.58 (0.49)	0.47 (0.50)	0.146
Bachelor Degree and above	0.49 (0.50)	0.42 (0.49)	0.53 (0.50)	0.146
**Ethnicity** (%)				
Other	0.29 (0.46)	0.35 (0.48)	0.27 (0.44)	0.109
White	0.71 (0.46)	0.65 (0.48)	0.73 (0.44)	0.109
**Income** (%)				
Less than $60,000	0.55 (0.50)	0.58 (0.49)	0.54 (0.50)	0.054
$60,000 and above	0.41 (0.49)	0.36 (0.48)	0.43 (0.50)	0.097
Prefer not to say	0.04 (0.19)	0.06 (0.24)	0.03 (0.17)	0.100
**Gender** (%)				
Male	0.42 (0.49)	0.40 (0.49)	0.44 (0.50)	0.052
Female	0.57 (0.50)	0.59 (0.49)	0.55 (0.50)	0.053
Other	0.01 (0.10)	0.01 (0.10)	0.01 (0.10)	0.001

Supplementary Table 6: **Comparing the demographic composition of Studies 1 and 2.** The sample of Study 1 is restricted to participants who were assigned to Saya's spectrum. The sample of Study 1′ is restricted to participants from Study 1 who did not respond to our invitation to participate in Study 2, while the sample of Study 2 is restricted to those who responded to our invitations and participated in Study 2. SMD stands for Standardized Mean Difference.

Supplementary Note 1: Spectrum Creation

There have been multiple attempts in the past to generate human faces that bypass the uncanny valley. One such attempt uses Progressively Growing Generative Adversarial Networks (PGGANs), which generate high-resolution human faces based on the CelebA dataset [1]. In contrast, our approach starts with an image that falls in the uncanny valley, and uses deep learning techniques to gradually take the image out of the valley by increasing its human resemblance. To this end, we use the StyleGAN model [2], which utilizes techniques from neural style transfer to allow for controlling various facial features in image synthesis. Examples of what could be done with such a model include the ability to morph the face of a person into that of their younger self, or to morph the face of a man into that of a woman [3]. A traditional generator architecture involves taking a random noise sample as an input and feeding that input through a series of convolutional and upsampling layers in order to generate an image. In contrast, the StyleGAN architecture takes a random noise sample and feeds it to a mapping network whose output is a noise sample that is not necessarily sampled from a Gaussian distribution; the mapping could be adjusted to any distribution of choice. Such a mapping network allows the output of the generator to be directly mapped to the input image vector, thereby allowing for gaps in the latent space where no data samples exist. As such, the StyleGAN architecture is based on manipulating images in the latent space rather than the pixel space. The process starts by finding a query image inside the latent space of the model. This problem in itself is seemingly intractable—given an image, would it be possible to find the latent code of that particular image inside the latent space such that it gives us the original image? Starting with a completely random latent vector, we obtain the feature vectors of both the query image as well the generator output and feed that to a pre-trained Convolutional Neural Network (CNN) such as the VGG-16 network [4] which is trained on a dataset such as ImageNet. Using these two feature vectors that include a high-level semantic representation of what is in the image, we can then calculate a simple loss function between these two vectors (such as the L2-loss) and back-propagate these gradients through the generator model given that the model is a fully differentiable neural network. However, this approach is quite slow because of the fact that we started with a completely random latent vector. In order to optimize the starting latent vector, we could sample a series of random noise to generate faces to compile a customized dataset. This dataset can then be trained on a second CNN such as a ResNet [5] to go from the images to the latent code. This trained ResNet can now be used on our query image to give us the best possible starting latent vector in order to obtain the best possible generated image. The entire process is summarized in Supplementary Figure 1.

The novelty of this approach lies in projecting the latent code of a query image into the StyleGAN's latent space, and using the ResNet to map any image to its latent code estimate. Since the generator in the StyleGAN has been trained to produce human faces, passing the latent code estimate through the generator gives us a very human-like face. The "Initial Guess" from Supplementary Figure 1 is the latent code that gives us a fully human face. However, we are attempting to create a spectrum of faces with varying degrees of "human-likeliness". By passing the generated image and the original query image through the VGG16 network, we can compute the L2-loss in the feature space and back-propagate to modify the original latent estimate. The image generated by this modified latent estimate is what produces an image that is somewhere between the fully human and the uncanny image. The criteria for selection was that the image had to be visibly "uncanny" upon a simple visual inspection, and the face should have a neutral facial expression in order to ensure there are no external factors influencing the decisions made our experiments. The only pre-processing step we take in this approach is ensuring that the background of the image is white to ensure that a background color does not get blended in with the image and to ensure uniformity across all the images. The generative models of the selected uncanny humanoid robots are illustrated in Supplementary Figures 2- 6.

Having used deep learning techniques to transform the uncanny image into a more human-like version, we wanted to use the same techniques to transform the image into a more machine-like version. Unfortunately, after multiple attempts, our deep learning-based approach has proven ineffective. Instead, we resorted to the use of a graphics editor—Photoshop—to manually edit the uncanny image and create multiple versions that are increasingly machine-like; see Supplementary Figures 7- 11).

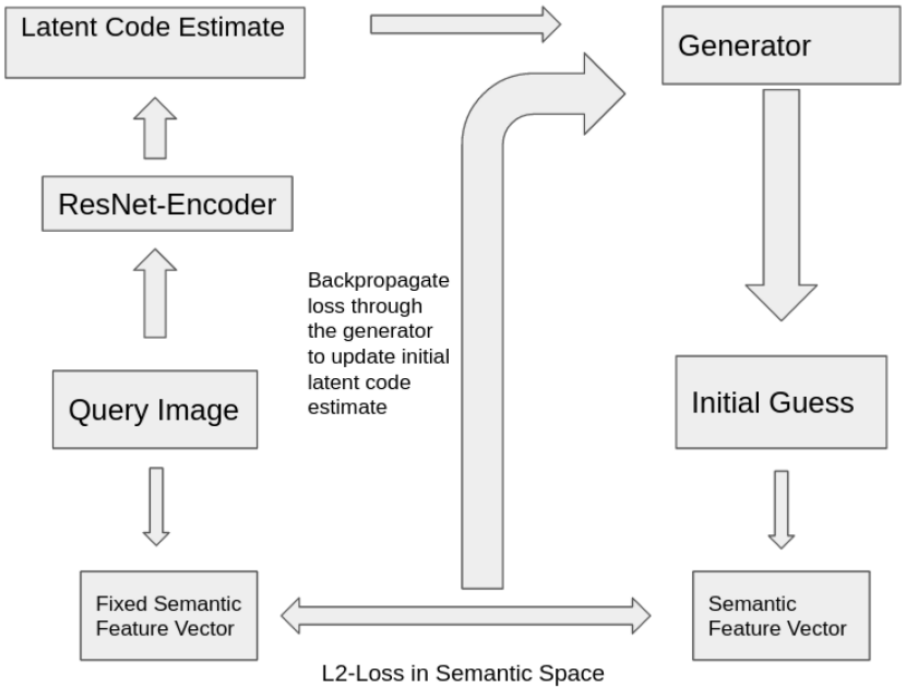

Supplementary Figure 1: **Image generator workflow.**

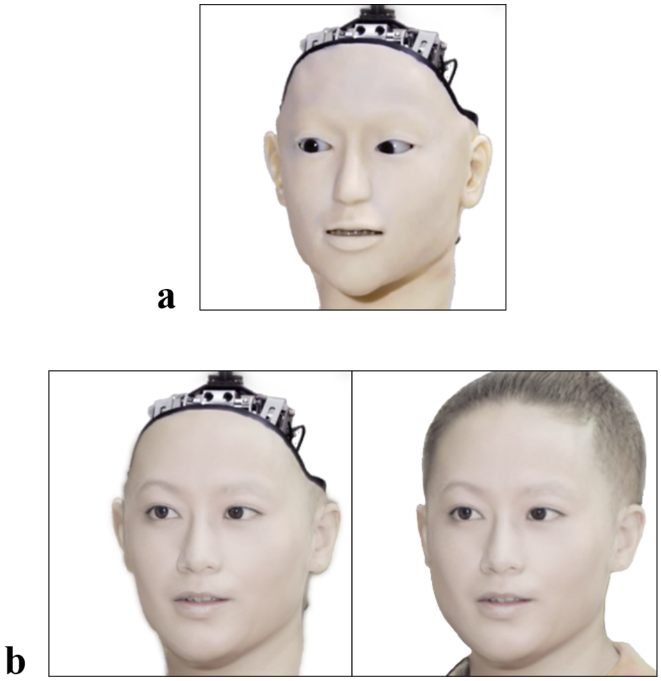

Supplementary Figure 2: Using StyleGAN on an original image of the robot **Alter** (a) to produce increasingly human-like versions (b).

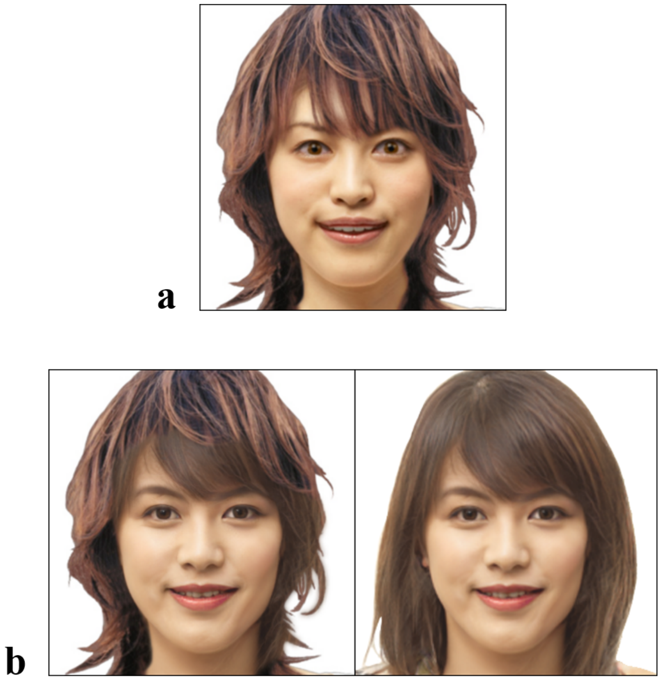

Supplementary Figure 3: Using StyleGAN on an original image of the CGI **Creepy Girl** (a) to produce increasingly human-like versions (b).

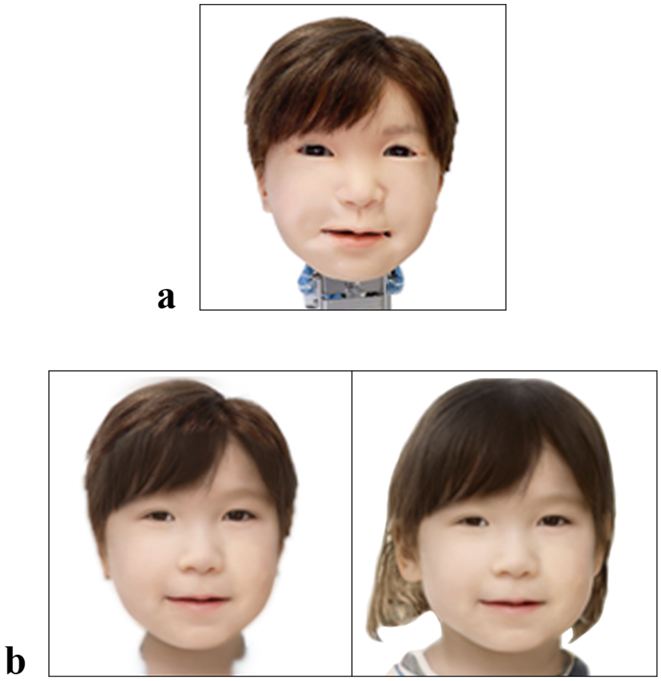

Supplementary Figure 4: Using StyleGAN on an original image of the robot **Affetto** (a) to produce increasingly human-like versions (b).

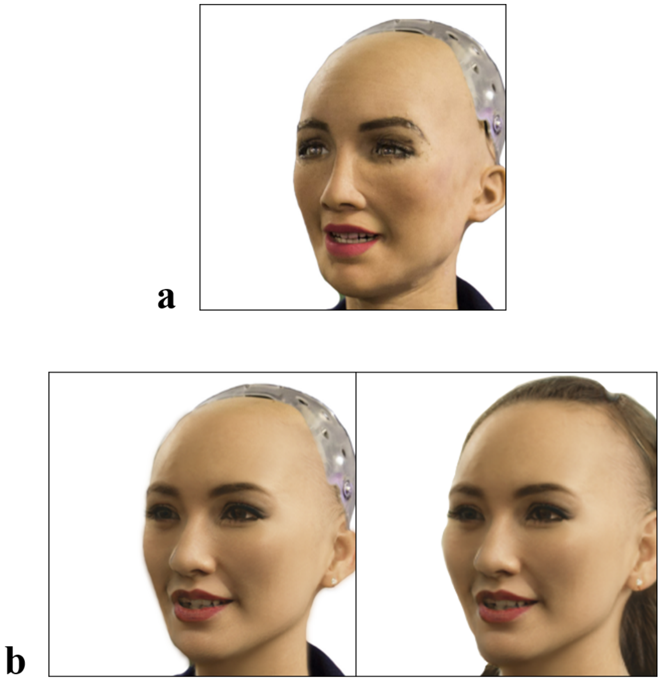

Supplementary Figure 5: Using StyleGAN on an original image of the robot **Sophia** (a) to produce increasingly human-like versions (b).

a

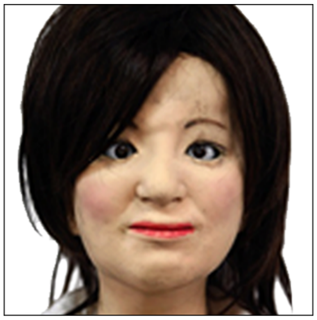

b

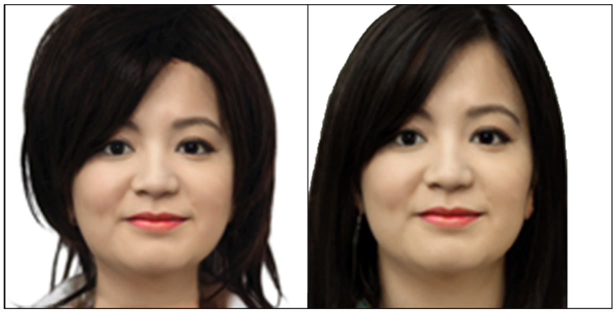

Supplementary Figure 6: Using StyleGAN on an original image of the robot **Saya** (a) to produce increasingly human-like versions (b).

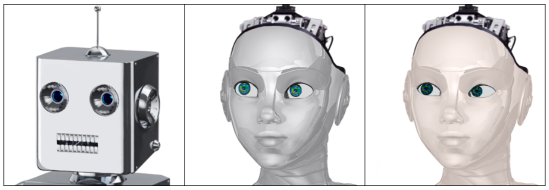

Supplementary Figure 7: Using Photoshop to create increasingly machine-like versions of **Alter**.

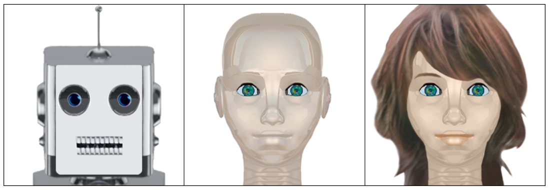

Supplementary Figure 8: Using Photoshop to create increasingly machine-like versions of **Creepy Girl**.

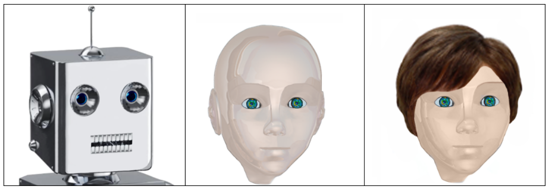

Supplementary Figure 9: Using Photoshop to create increasingly machine-like versions of **Affetto**.

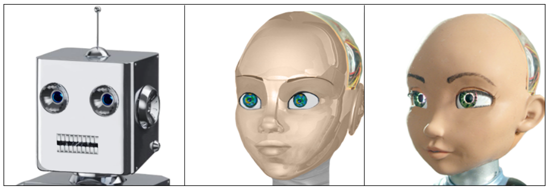

Supplementary Figure 10: Using Photoshop to create increasingly machine-like versions of **Sophia**.

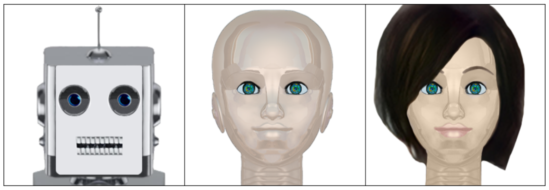

Supplementary Figure 11: Using Photoshop to create increasingly machine-like versions **Saya**.

Supplementary Note 2: Spectrum Evaluation

The user interface of Study 1 is depicted in Supplementary Figure 12. When measuring "pleasantness," we relied on the users' responses to the question: "*How pleasant would it be to interact with the figure*

represented by this image?". One could argue that an alternative measure of pleasantness could be the users' responses to the question: "*How do you feel toward the figure represented by this image?*". However, the users' responses to both questions are very strongly correlated (r =0.9), implying that they can be used interchangeably. Given this strong correlation, we decided to only use the former in our analysis.

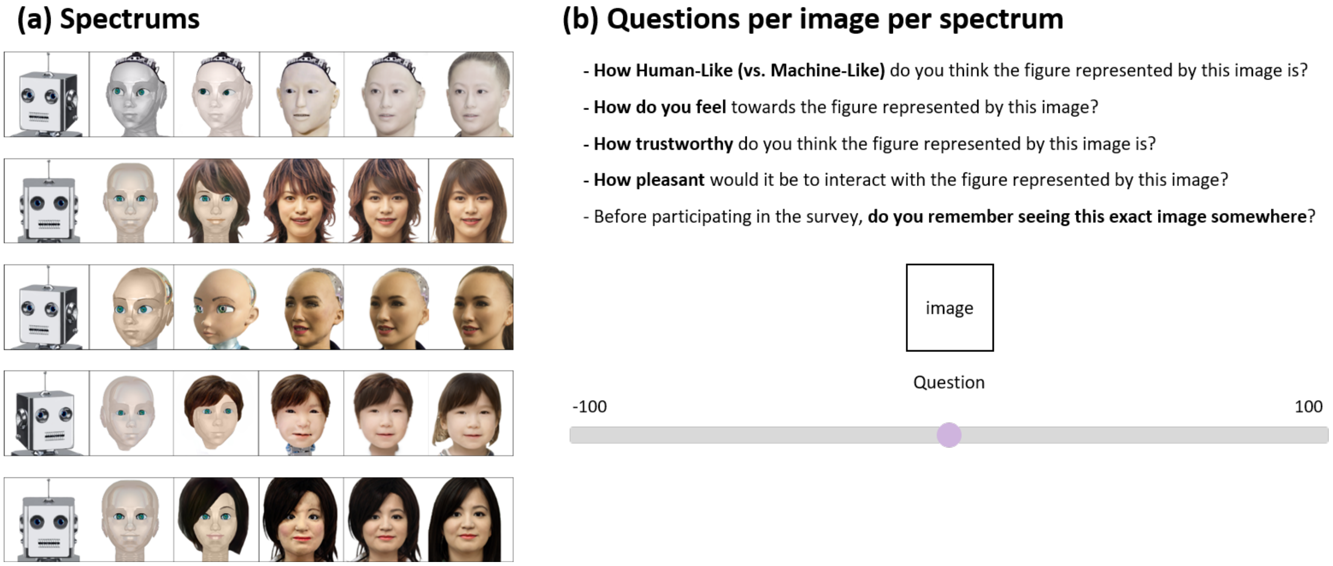

Supplementary Figure 12: **Study 1 survey questions.** Each participant is randomly assigned to one of the designed spectra (a) and then asked a set of questions, for each image along the spectrum. Each question is asked separately in a dedicated page (b).

Supplementary Note 3: Study 2 - Graphical User Interface (GUI)

As part of this study you will complete **three activities**. For each activity, you will be provided with instructions, and then you will answer three comprehension check questions. Following this, you will make decisions that will impact the amount of money you can earn as a bonus in addition to your $1.50 as a show-up fee. Furthermore, upon the completion of the data collection of this study, five participants will be chosen randomly to win a $50.00 Amazon Gift Card.

Note that, for each activity, if you answer the comprehension check questions correctly on your first attempt, you will receive a bonus of $0.25. Thus, you may earn a total of $0.75 for answering the comprehension check questions correctly for all three activities on your first attempt in addition to a maximum of $5.00 bonus from the three activities.

You will be paired with the **same set of six Associates** in all three activities, however, **only one** of the six interactions per activity will be randomly selected for payment. Furthermore, your decisions will only be communicated to your Associates after you have made all of your decisions. For example, your decision in the first activity will not be communicated until all decisions have been made in the first, second, and third activities.

All of your Associates are robots that have been programmed to interact socially with humans and make decisions in these activities.

Supplementary Figure 13: **Study 2 GUI – Introduction.**

Activity 1

As part of this activity, you will have the opportunity to earn up to $3.00. This is in addition to the $1.00 you will receive for completing this study. Your earnings will depend on the decision you and your Associates make in this activity. So, we encourage you to read and follow all instructions carefully.

After you finish reading the instructions, you will be asked some comprehension questions to verify that you understood them. You can only participate in the study once you have answered **all questions** correctly. If you do not get all of the comprehension check questions right, you will have an opportunity to review the instructions, and answer them again, until you answer them all correctly. If you answer the comprehension check questions correctly on the first attempt, you will earn $0.25 as a bonus.

You are going to do this activity with an Associate. At the start of this activity, you receive $1,00 and **you get to choose how much of this $1.00, if any, to send to your Associate**. **Any amount you send to your Associate is tripled**. In other words, for every cent that you send, your associate receives three cents.

Then, **your Associate chooses how much out of the received amount, if any, to return to you**. Your Associate can send all, some, or none back to you.

Example: Suppose you sent the entire $1.00 to your Associate. Then, your Associate will receive $3.00. Now, if your Associate returns half of the $3.00, you will earn $1.50, ending up with more than the $1.00 you started with. However, if your Associate returns nothing, you will end up with less than the $1.00 you started with. So, **you can gain or lose by sending money to your Associate, depending on how much they return**.

Supplementary Figure 14: **Study 2 GUI – Description of Trust Game.**

The illustration below summarizes the activity. As can be seen from the illustration, **your bonus** is the amount you keep plus the amount that your Associate sends back. **Your Associate's bonus** is three times the amount you send minus the amount they send back.

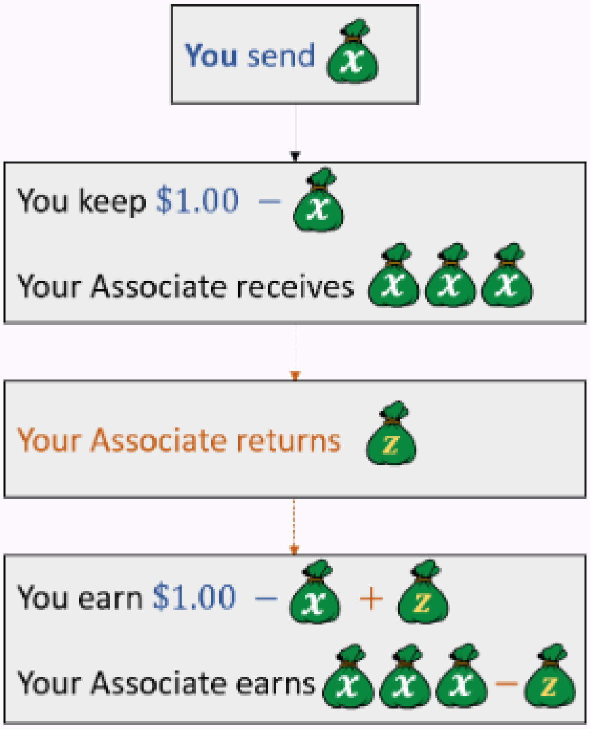

Supplementary Figure 15: **Study 2 GUI – Illustration of Trust Game.**

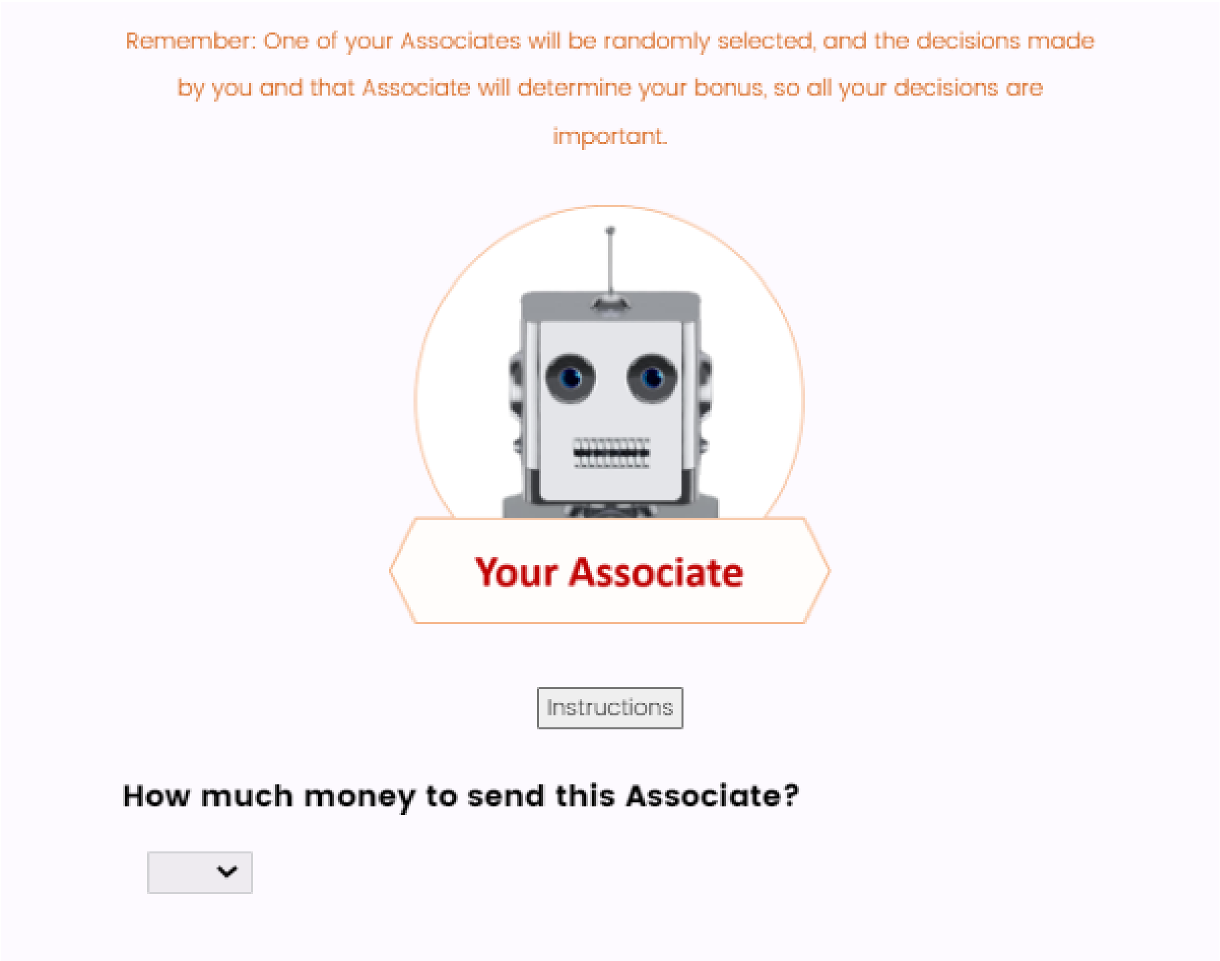

Supplementary Figure 16: **Study 2 GUI – Decision making in Trust Game.** For each of the six associates, participants select the amount of money to send to that associate. Here, associates are displayed in random order.

Activity 2

As part of this activity, you will have the opportunity to earn up to $1.00. This is in addition to the $1.00 you will receive for completing this study. Your earnings will depend on the decision that you make in this activity. So, we encourage you to read and follow all instructions carefully.

After you finish reading the instructions, you will be asked a set of comprehension check questions to verify that you understood them. **You can only participate in the study once you have answered all questions correctly.** If you do not get all comprehension check questions right, you will have an opportunity to review the instructions, and answer them again, until you have answered them all correctly. If you answer all questions correctly on the first attempt, you will earn $0.25 as a bonus.

You are going to do this activity with an Associate. At the start of this activity, you will receive $1.00. Then, you will have to decide how much money, if any, to send to your Associate. You can send all, some, or none of the $1.00 to your Associate.

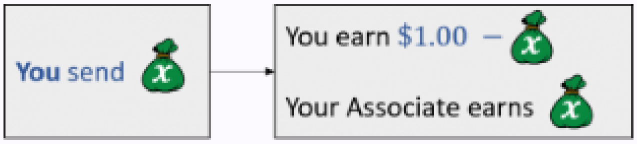

Your bonus from this activity is the amount you keep from the $1.00. Your Associate's bonus from this activity is the amount you send them from the $1.00.

Supplementary Figure 17: **Study 2 GUI – Description of Dictator Game.**.

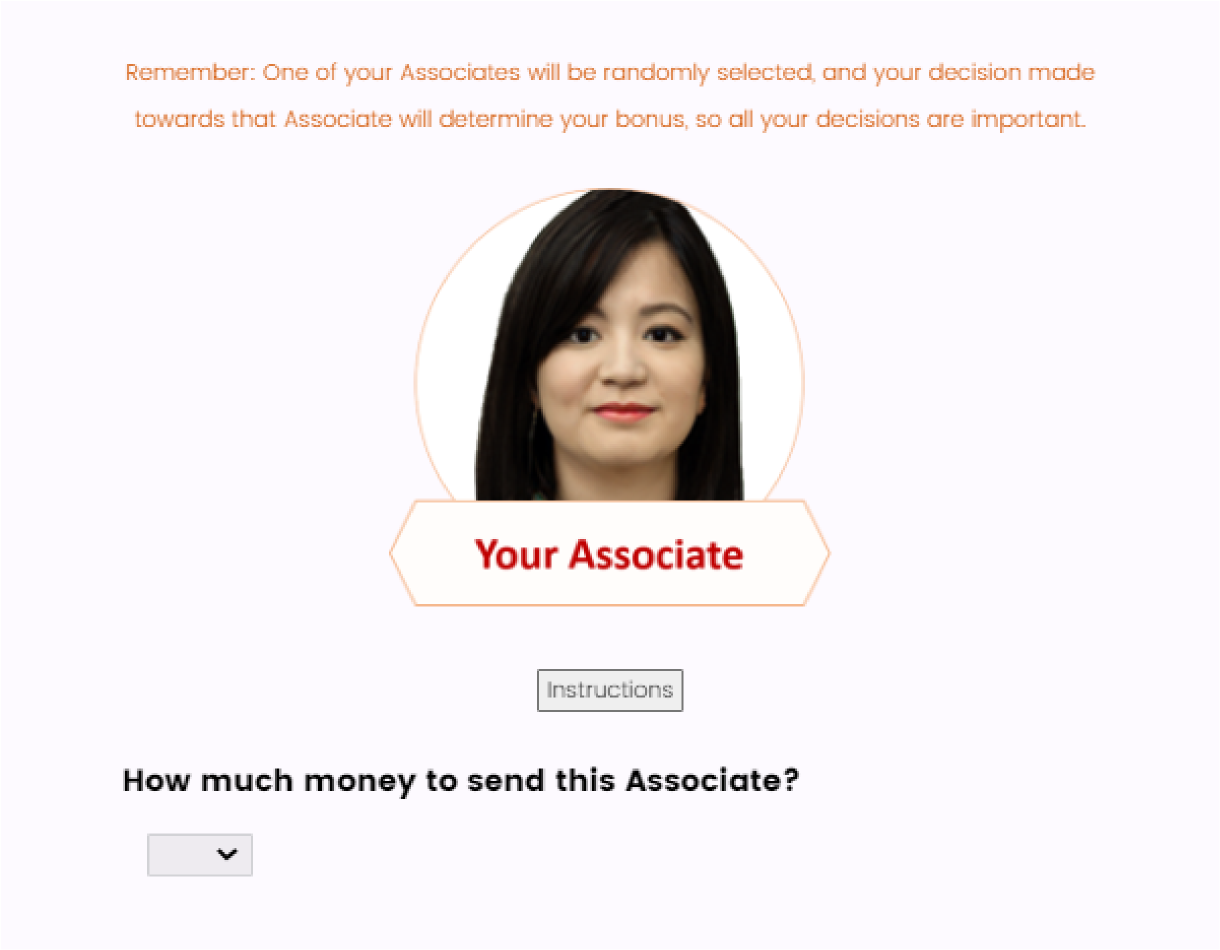

Supplementary Figure 18: **Study 2 GUI – Decision making in Dictator Game.** For each of the six associates, participants select the amount of money to send to that associate. Here, associates are displayed in random order.

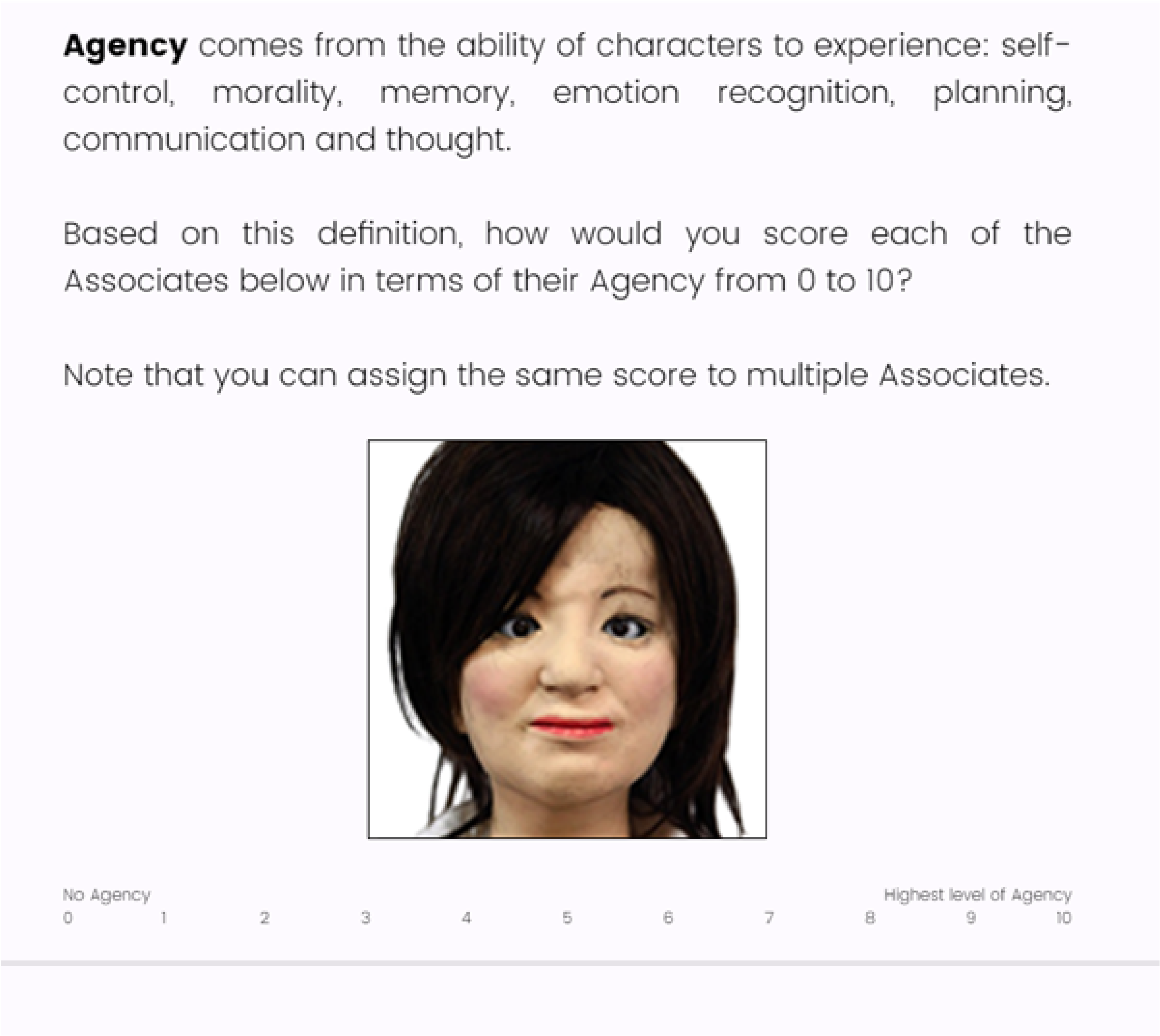

Supplementary Figure 19: **Study 2 GUI – Evaluating associates' agency.** Participants assign a level of Agency on a slider (from 0 to 10) for each of the six associates. Here, associates are displayed in random order.

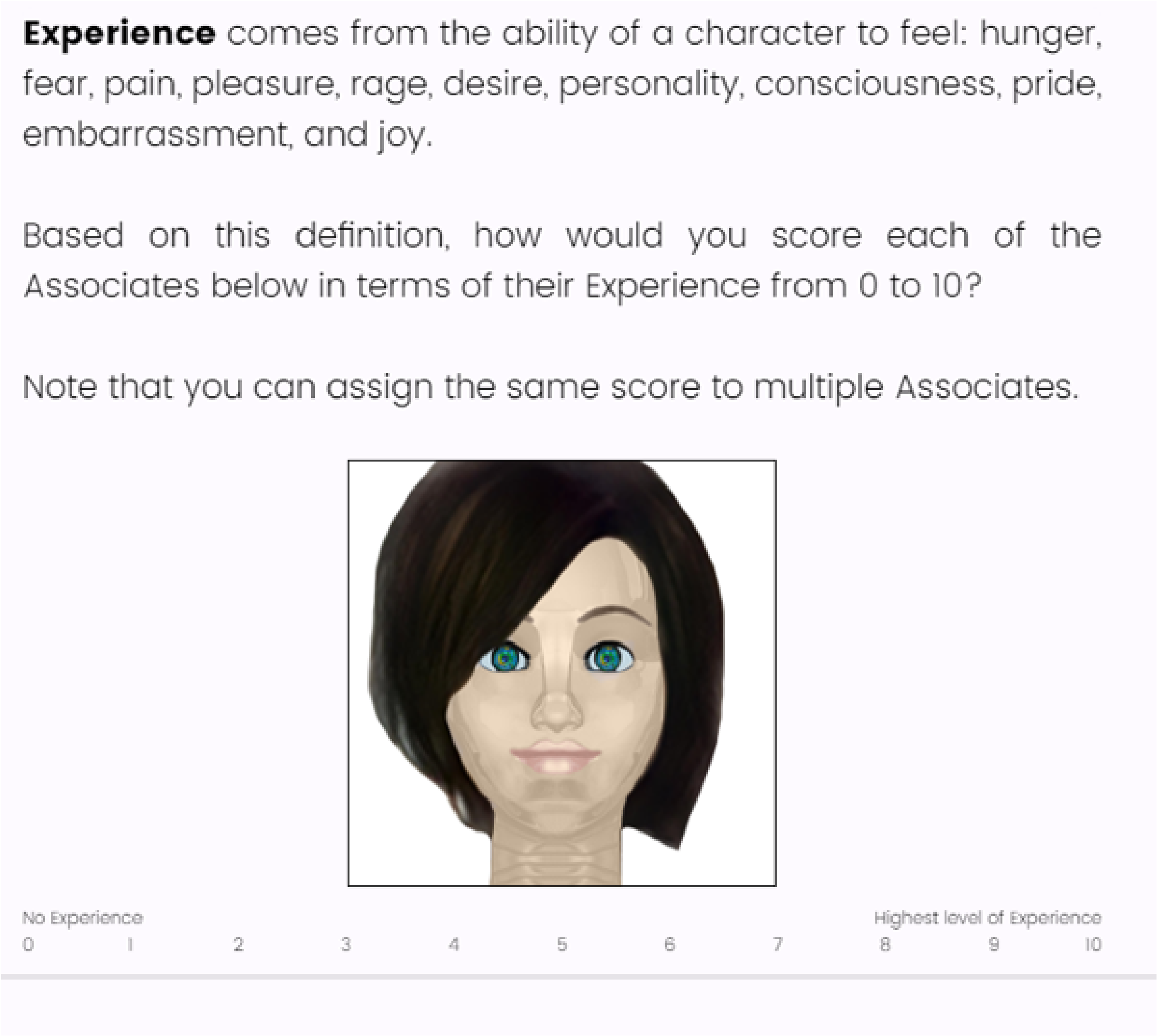

Supplementary Figure 20: **Study 2 GUI – Evaluating associates' experience.** Participants assign a level of Agency on a slider (from 0 to 10) for each of the six associates. Here, associates are displayed in random order.

Supplementary Note 4: Study 3 - Graphical User Interface (GUI)

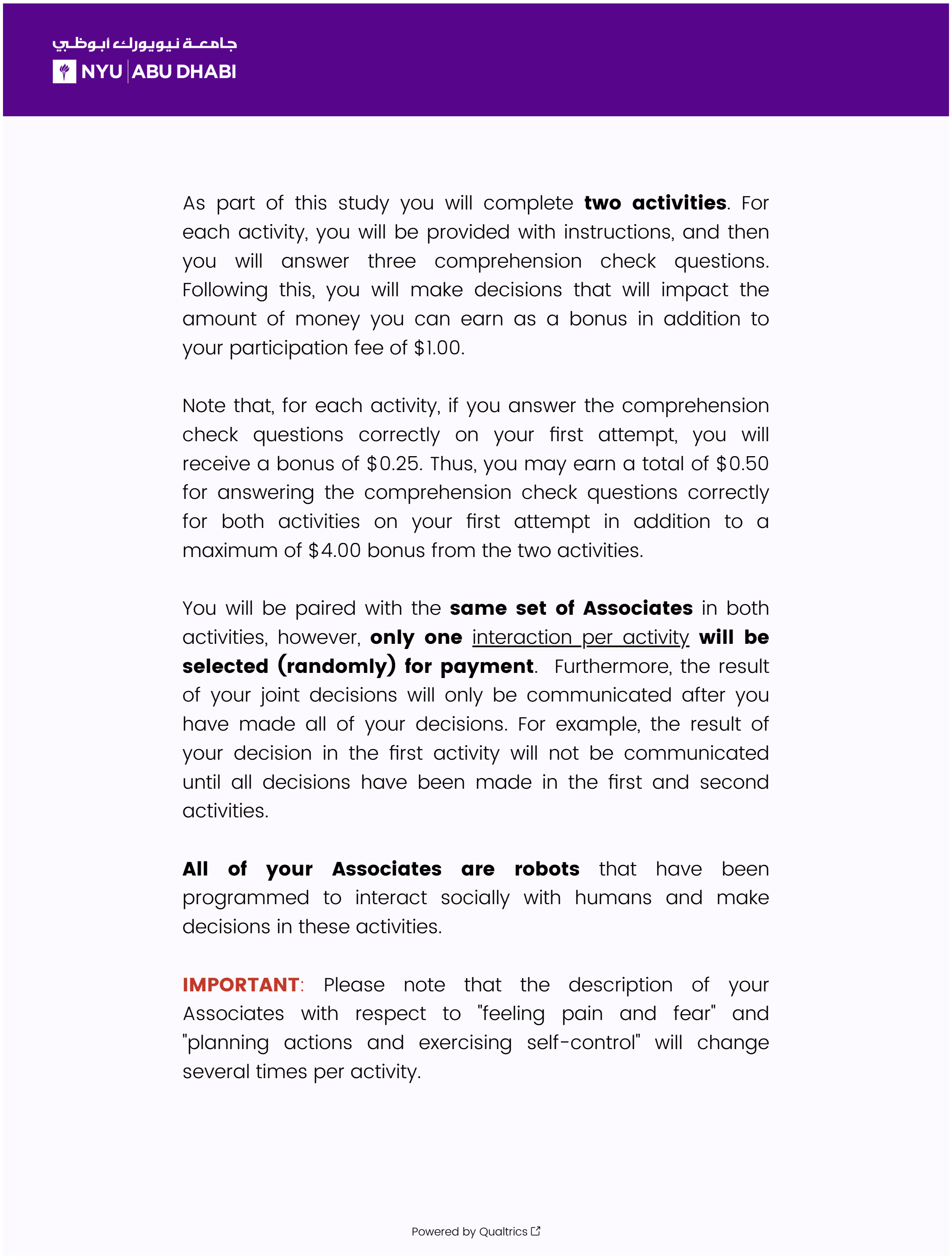
جامعة نيويورك ابوظبي
NYU | ABU DHABI

As part of this study you will complete **two activities**. For each activity, you will be provided with instructions, and then you will answer three comprehension check questions. Following this, you will make decisions that will impact the amount of money you can earn as a bonus in addition to your participation fee of $1.00.

Note that, for each activity, if you answer the comprehension check questions correctly on your first attempt, you will receive a bonus of $0.25. Thus, you may earn a total of $0.50 for answering the comprehension check questions correctly for both activities on your first attempt in addition to a maximum of $4.00 bonus from the two activities.

You will be paired with the **same set of Associates** in both activities, however, **only one** interaction per activity **will be selected (randomly) for payment**. Furthermore, the result of your joint decisions will only be communicated after you have made all of your decisions. For example, the result of your decision in the first activity will not be communicated until all decisions have been made in the first and second activities.

All of your Associates are robots that have been programmed to interact socially with humans and make decisions in these activities.

IMPORTANT: Please note that the description of your Associates with respect to "feeling pain and fear" and "planning actions and exercising self-control" will change several times per activity.

Powered by Qualtrics

Supplementary Figure 21: **Study 3 GUI – Introduction.** The description of the two activities matches that of Study 2.

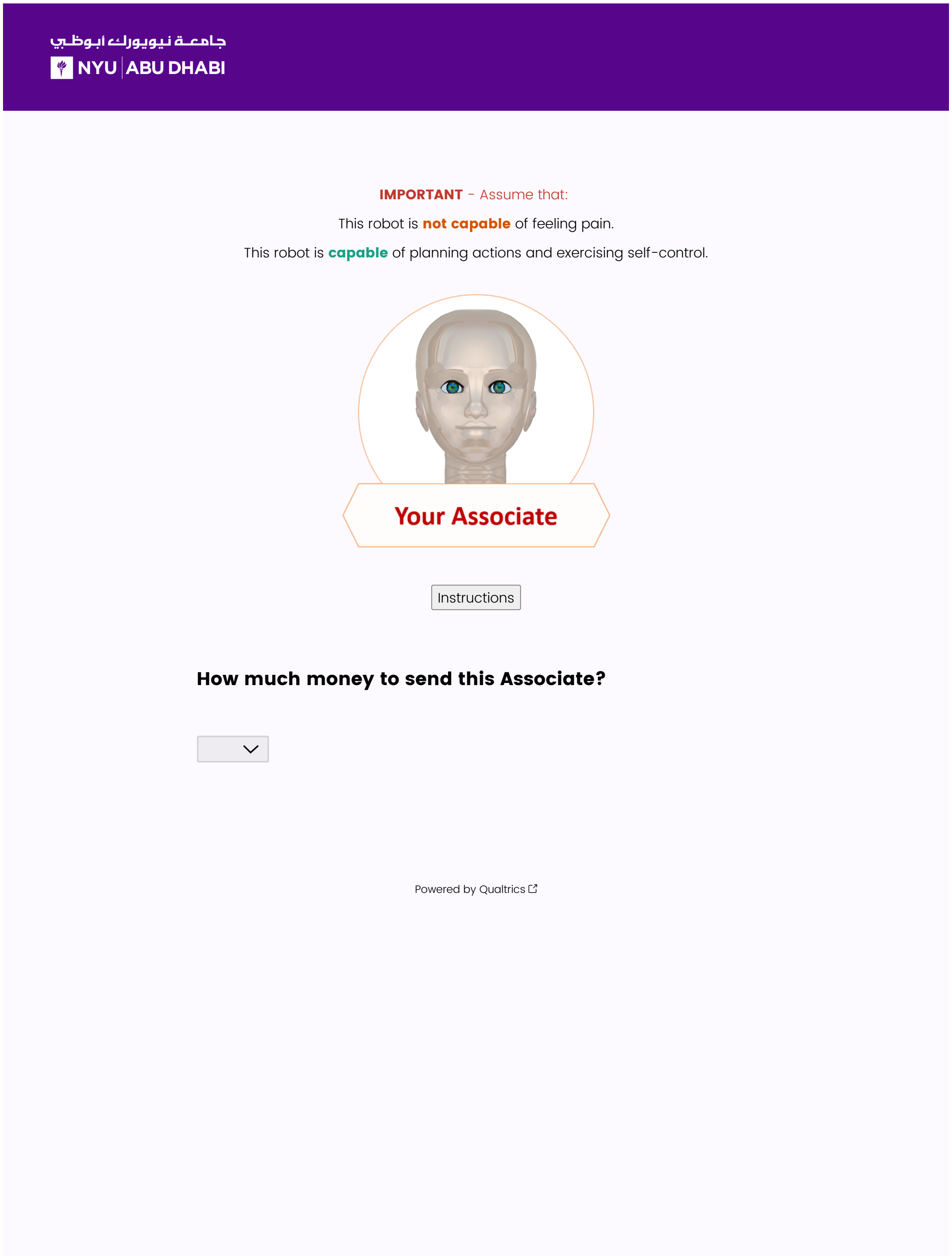

Supplementary Figure 22: **Study 3 GUI – Making a decision in an Activity with the first bot image under the condition of "agency but no experience."** The description of the robot varies depending on the condition. Each participant interacts with all 4 conditions per bot image randomly.

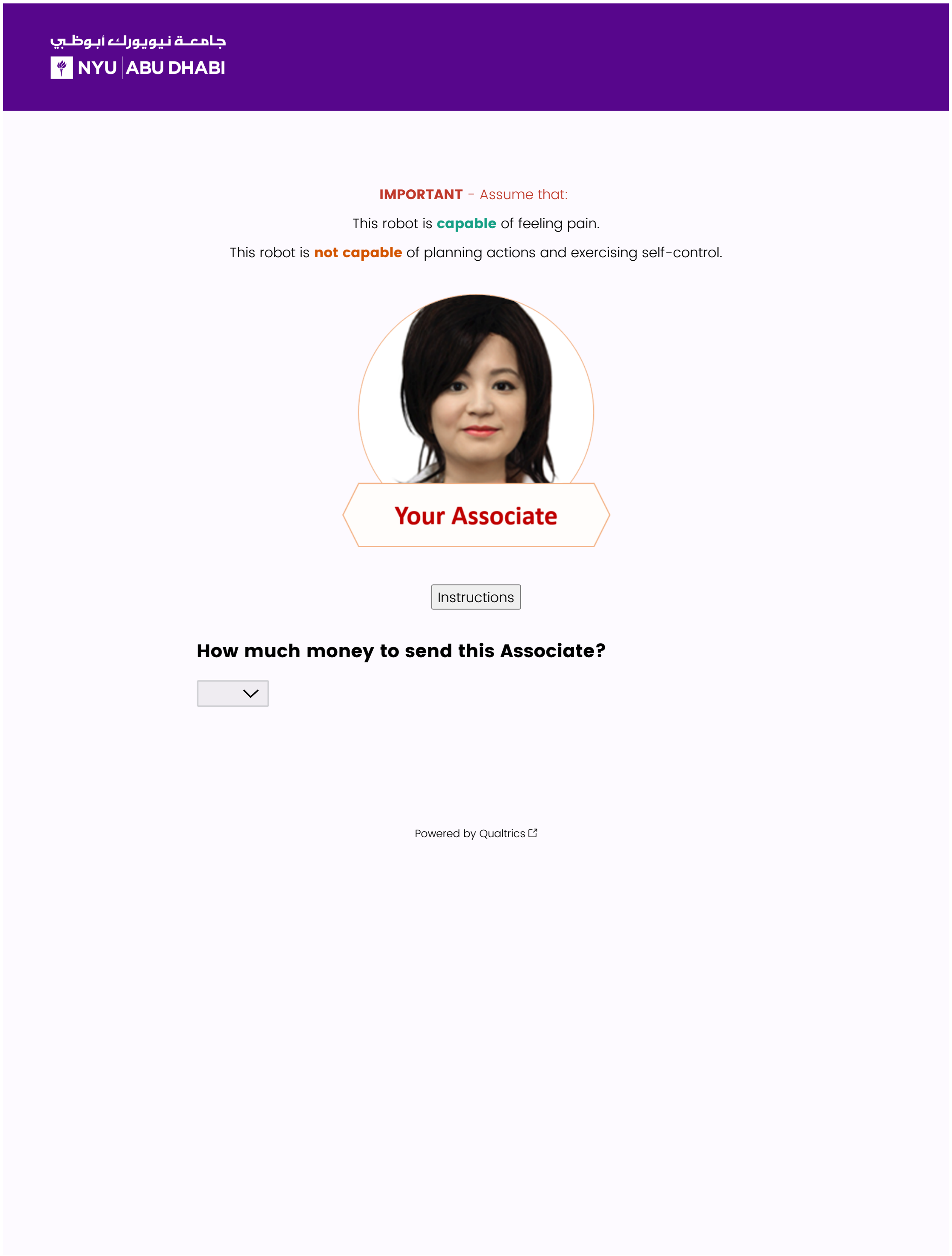

Supplementary Figure 23: **Study 3 GUI – Making a decision in an Activity with the second bot image under the condition of "experience but no agency."** The description of the robot varies depending on the condition. Each participant interacts with all 4 conditions per bot image randomly.

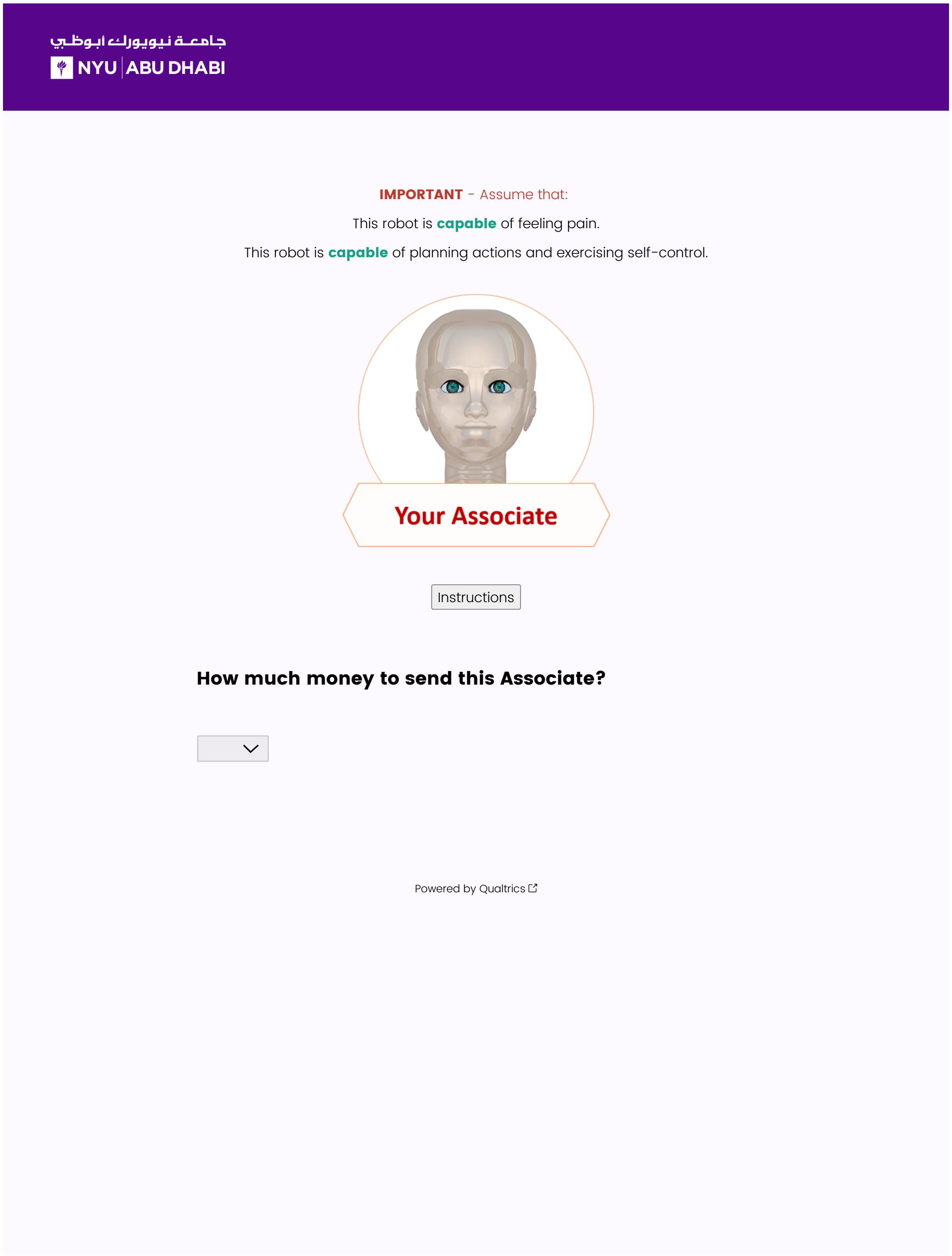

Supplementary Figure 24: **Study 3 GUI – Making a decision in an Activity with the first bot image under the condition of "agency and experience."** The description of the robot varies depending on the condition. Each participant interacts with all 4 conditions per bot image randomly.

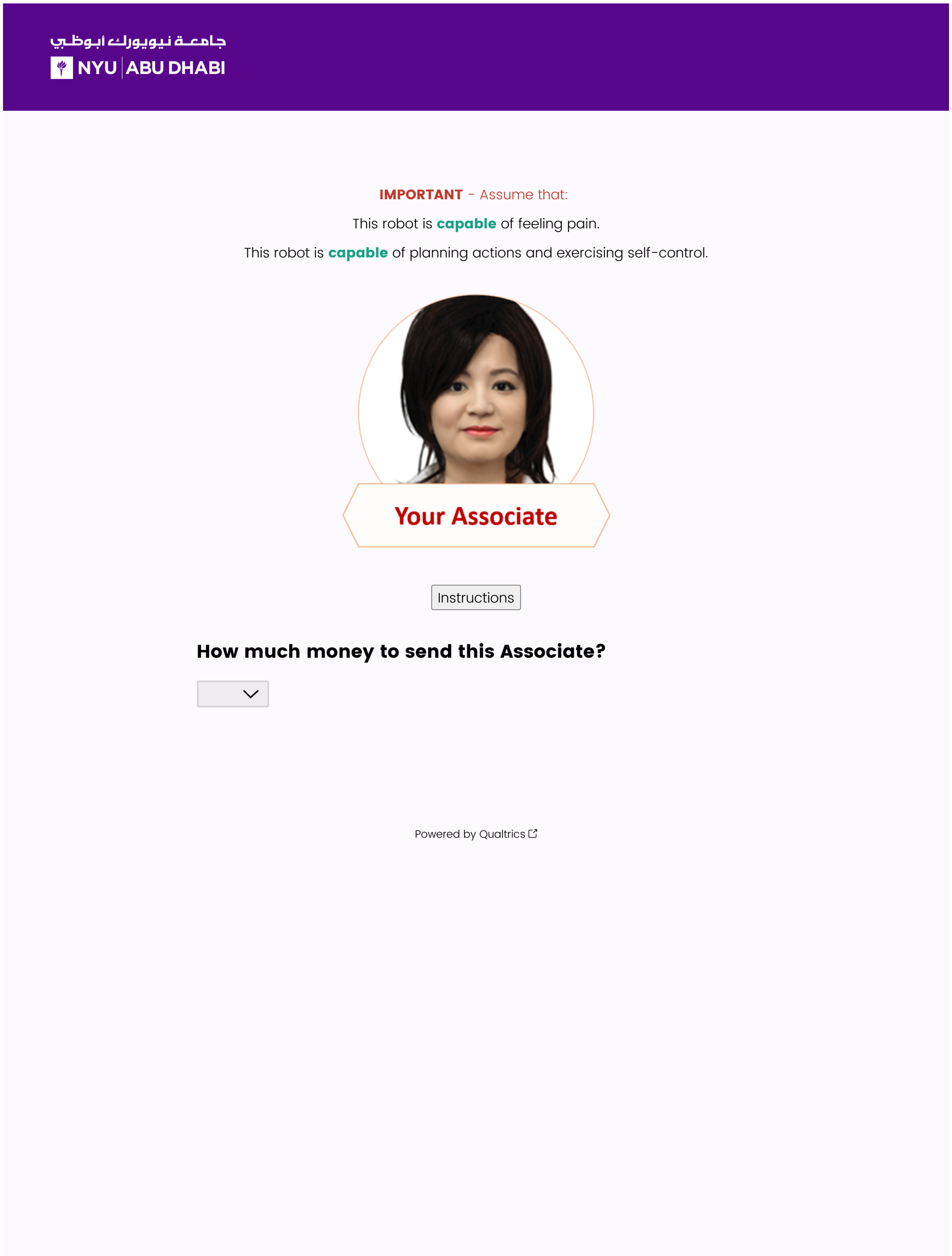

Supplementary Figure 25: **Study 3 GUI – Making a decision in an Activity with the second bot image under the condition of "agency and experience."** The description of the robot varies depending on the condition. Each participant interacts with all 4 conditions per bot image randomly.

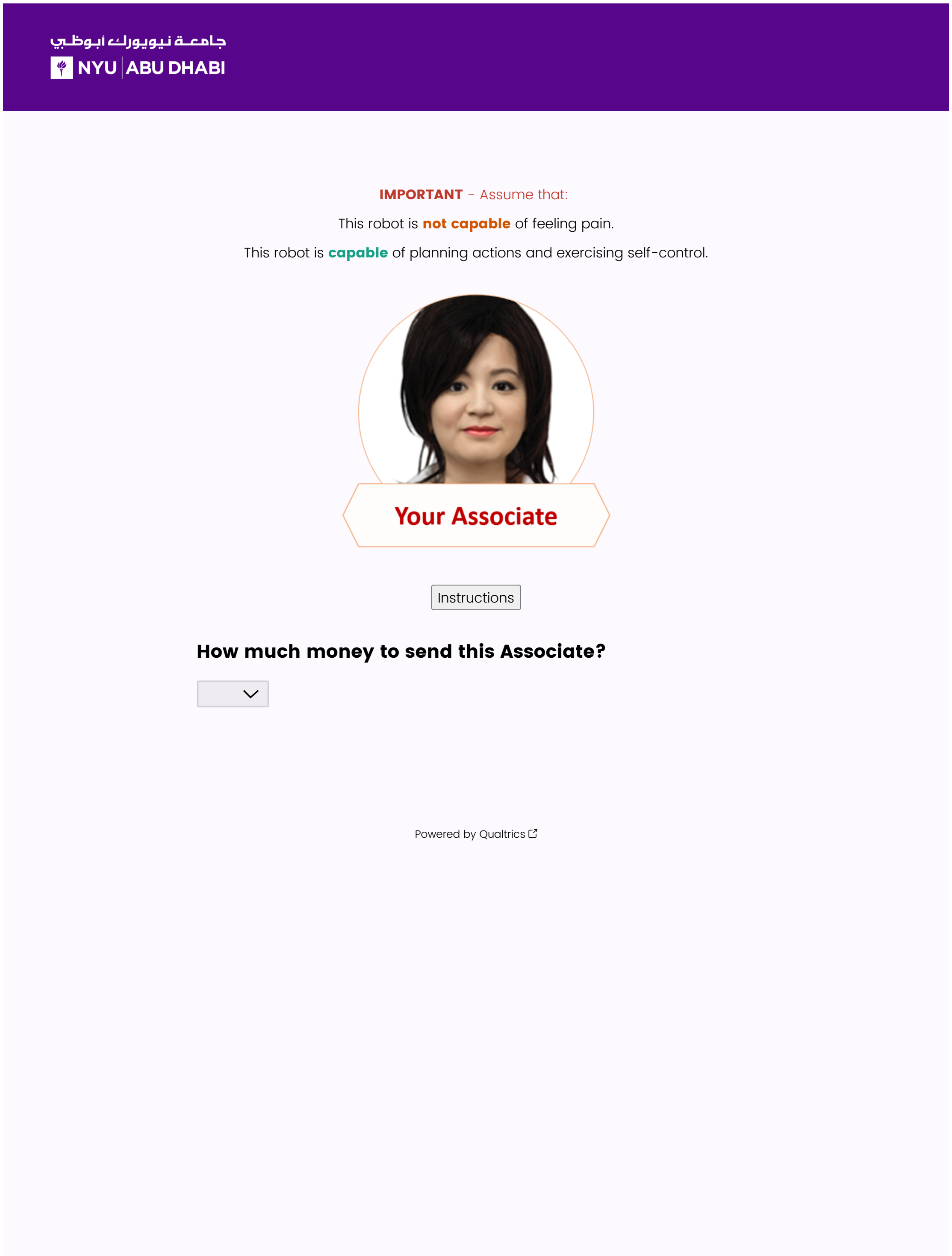

Supplementary Figure 26: **Study 3 GUI – Making a decision in an Activity with the second bot image under the condition of "agency but no experience."** The description of the robot varies depending on the condition. Each participant interacts with all 4 conditions per bot image randomly.

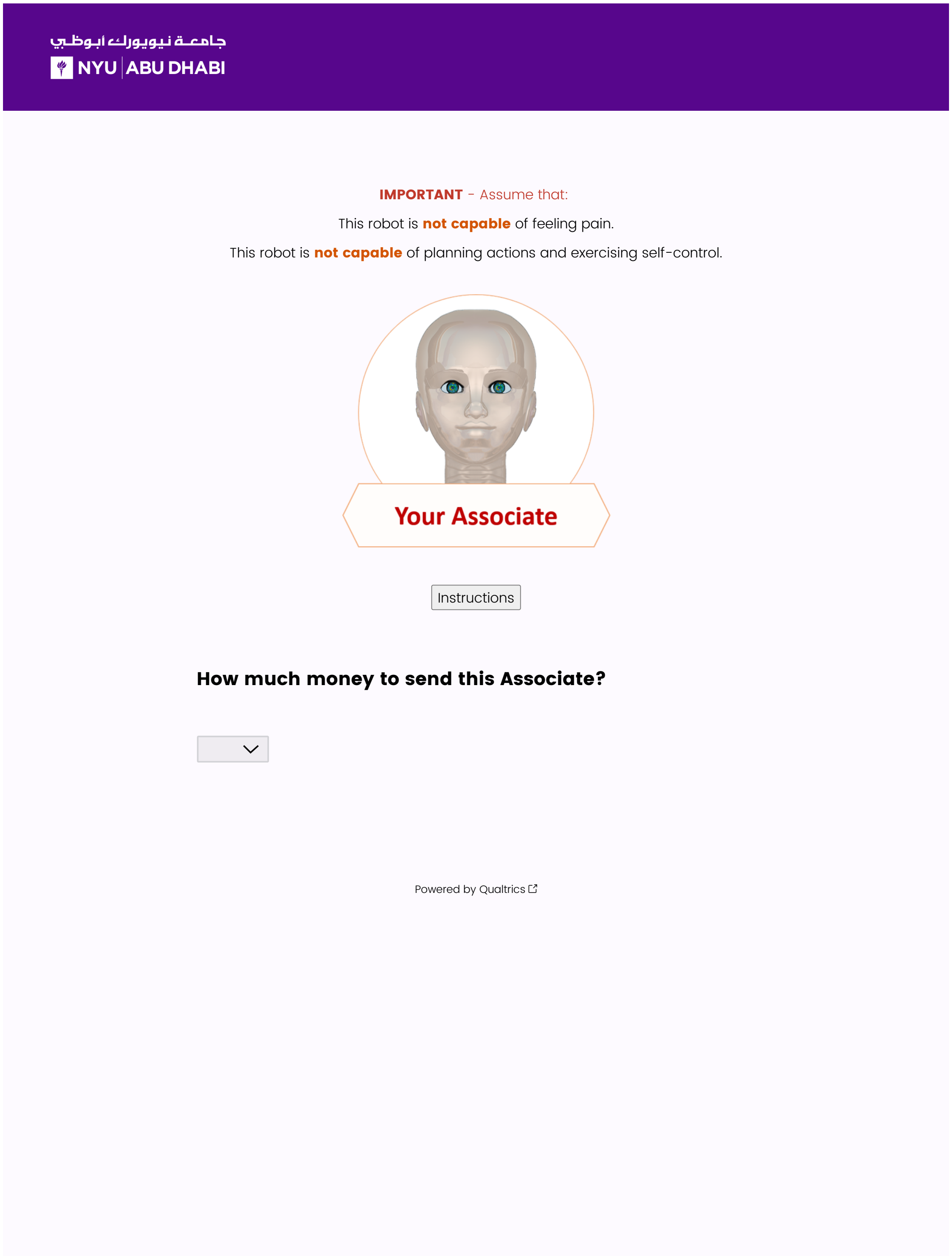

Supplementary Figure 27: **Study 3 GUI – Making a decision in an Activity with the first bot image under the condition of "no agency nor experience."** The description of the robot varies depending on the condition. Each participant interacts with all 4 conditions per bot image randomly.

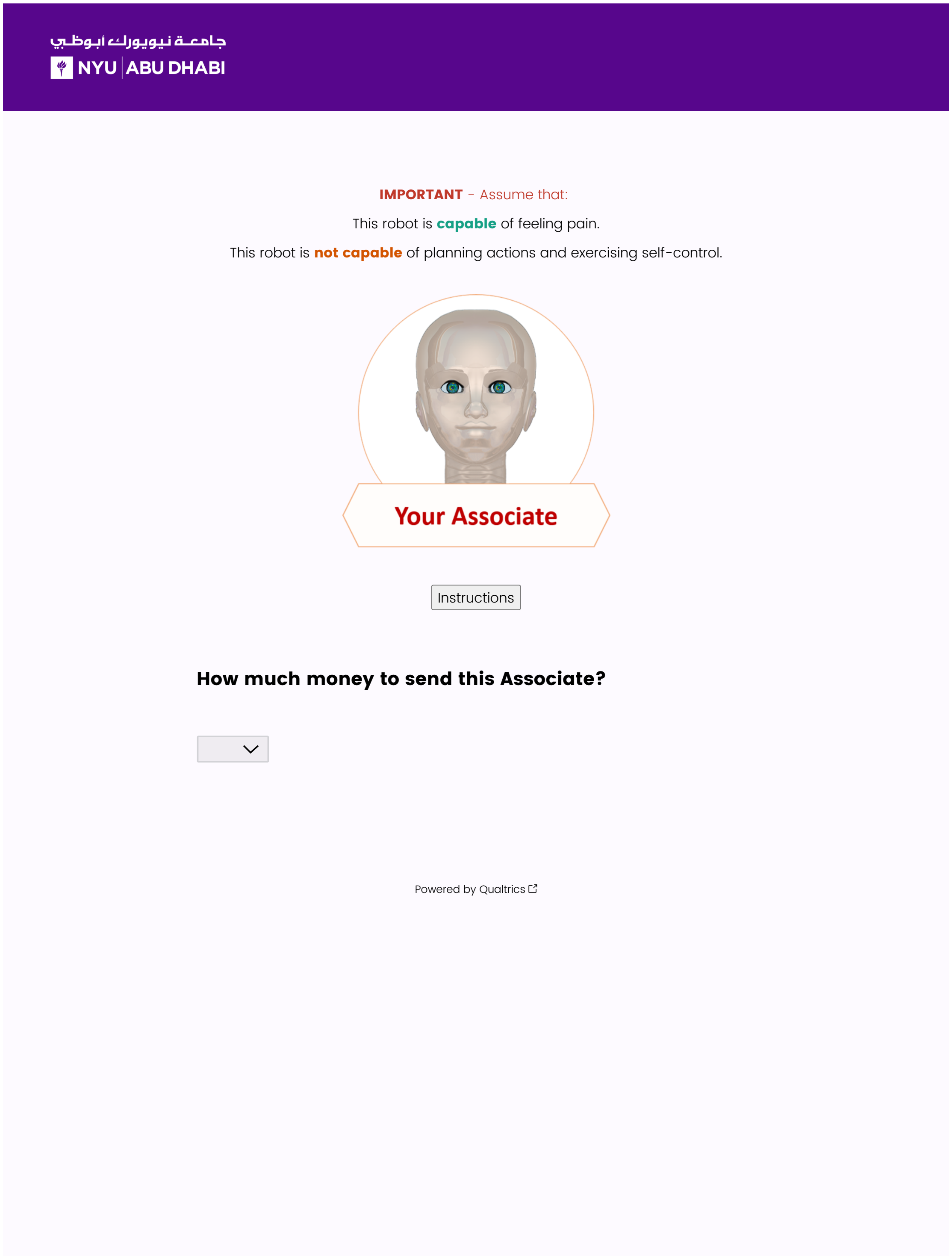

Supplementary Figure 28: **Study 3 GUI – Making a decision in an Activity with the first bot image under the condition of "experience but no agency."** The description of the robot varies depending on the condition. Each participant interacts with all 4 conditions per bot image randomly.

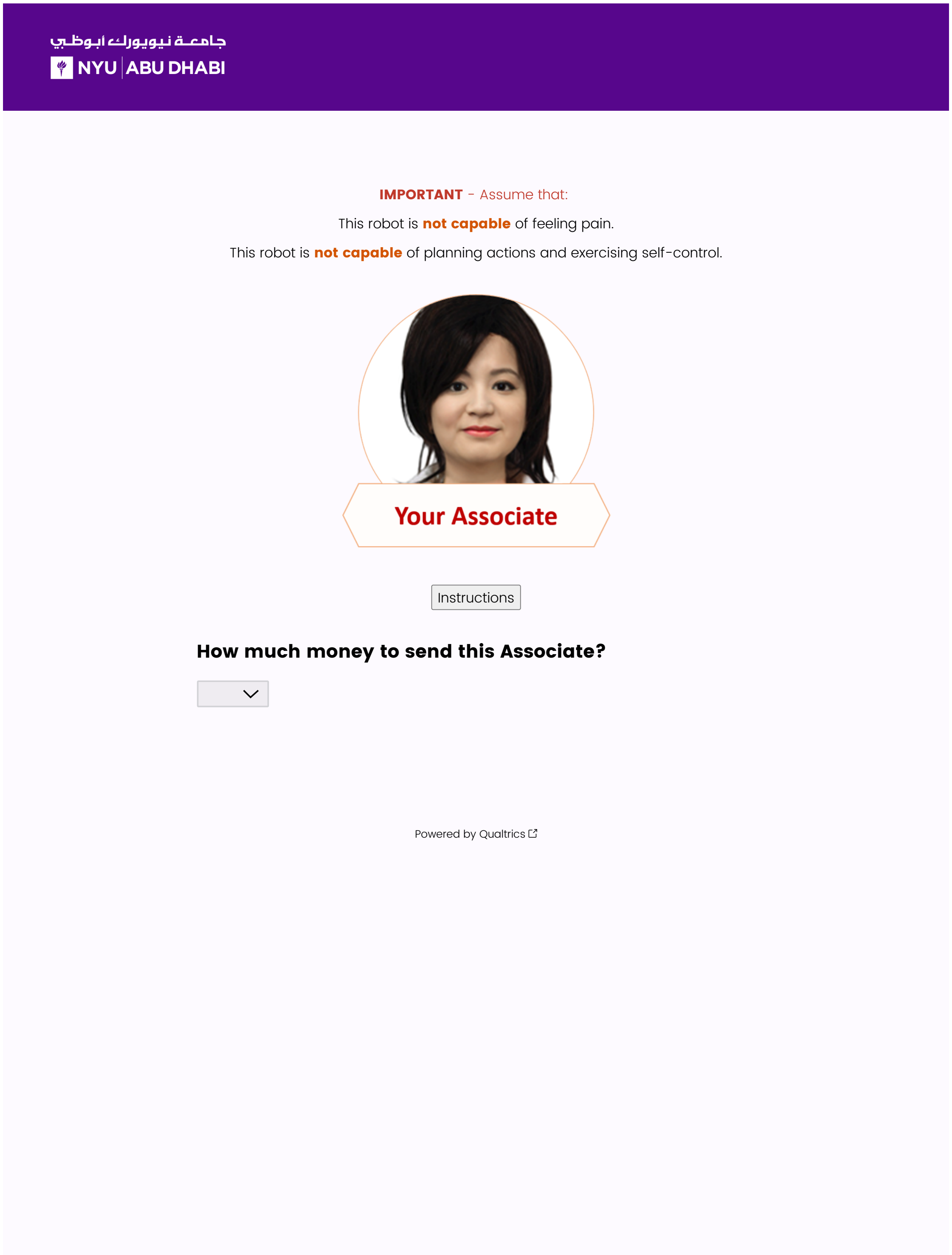

Supplementary Figure 29: **Study 3 GUI – Making a decision in an Activity with the second bot image under the condition of "no agency nor experience."** The description of the robot varies depending on the condition. Each participant interacts with all 4 conditions per bot image randomly.

References

[1] Karras, T., Aila, T., Laine, S. & Lehtinen, J. Progressive growing of GANs for improved quality, stability, and variation. In International Conference on Learning Representations (2018). URL https://openreview.net/forum?id=Hk99zCeAb.

[2] Karras, T., Laine, S. & Aila, T. A style-based generator architecture for generative adversarial networks. In IEEE/CVF Conference on Computer Vision and Pattern Recognition (CVPR), 4396–4405 (2019).

[3] Baylies, P. Stylegan-encoder: Stylegan encoder - converts real images to latent space (2019). URL https://github.com/pbaylies/stylegan-encoder.

[4] Simonyan, K. & Zisserman, A. Very Deep Convolutional Networks for Large-Scale Image Recognition. arXiv e-prints arXiv:1409.1556 (2014). 1409.1556.

[5] He, K., Zhang, X., Ren, S. & Sun, J. Deep residual learning for image recognition. In 2016 IEEE Conference on Computer Vision and Pattern Recognition (CVPR), 770–778 (2016).
